# Modeling the dependence of respiration and photosynthesis upon light, acetate, carbon dioxide, nitrate and ammonium in *Chlamydomonas reinhardtii* using design of experiments and multiple regression

**DOI:** 10.1186/s12918-014-0096-0

**Published:** 2014-08-16

**Authors:** Stéphanie Gérin, Grégory Mathy, Fabrice Franck

**Affiliations:** 1 Laboratory of Bioenergetics, Department of Life Sciences, Faculty of Sciences, University of Liege, Boulevard du Rectorat 27, Liege, 4000, Belgium; 2 Cell Culture Process Sciences, UCB Pharma, Avenue de l’Industrie, Braine l’Alleud, 1420, Belgium

**Keywords:** Statistics, Bioenergetics, Metabolism, Network, Plasticity, Environment

## Abstract

**Background:**

In photosynthetic organisms, the influence of light, carbon and inorganic nitrogen sources on the cellular bioenergetics has extensively been studied independently, but little information is available on the cumulative effects of these factors. Here, sequential statistical analyses based on design of experiments (DOE) coupled to standard least squares multiple regression have been undertaken to model the dependence of respiratory and photosynthetic responses (assessed by oxymetric and chlorophyll fluorescence measurements) upon the concomitant modulation of light intensity as well as acetate, CO_2_, nitrate and ammonium concentrations in the culture medium of *Chlamydomonas reinhardtii*. The main goals of these analyses were to explain response variability (i.e. bioenergetic plasticity) and to characterize quantitatively the influence of the major explanatory factor(s).

**Results:**

For each response, 2 successive rounds of multiple regression coupled to one-way ANOVA *F*-tests have been undertaken to select the major explanatory factor(s) (1st-round) and mathematically simulate their influence (2nd-round). These analyses reveal that a maximal number of 3 environmental factors over 5 is sufficient to explain most of the response variability, and interestingly highlight quadratic effects and second-order interactions in some cases. In parallel, the predictive ability of the 2nd-round models has also been investigated by *k*-fold cross-validation and experimental validation tests on new random combinations of factors. These validation procedures tend to indicate that the 2nd-round models can also be used to predict the responses with an inherent deviation quantified by the analytical error of the models.

**Conclusions:**

Altogether, the results of the 2 rounds of modeling provide an overview of the bioenergetic adaptations of *C. reinhardtii* to changing environmental conditions and point out promising tracks for future in-depth investigations of the molecular mechanisms underlying the present observations.

## Background

In plants and algae, energy transduction processes involve the respiratory and photosynthetic electron transport chains, which take place at the level of the mitochondrial inner membrane and the thylakoid, respectively, through chemi-osmotic mechanisms coupling electron transport and ADP phosphorylation [1]. An ubiquinol-O_2_ terminal oxidase (alternative oxidase, AOX) which competes with complex III for electrons is also found in the mitochondrial inner membrane and is responsible for a cyanide-insensitive “alternative” respiratory pathway, opposed to the “cytochromial” pathway due to complexes III and IV. AOX activity does not contribute to the building of the electrochemical proton gradient and is therefore qualified as “energy-dissipating”. This enzyme has long been known to be responsible for heat production in the thermogenic tissues of higher plants (the spadix of *Araceae*) but is also thought to play important roles in non-thermogenic cells in some circumstances by limiting the production of superoxide anion by complexes I and III and accelerating the turnover of reduced cofactors to ensure a continuous operation of the primary metabolism [2].

Over the last century, efficient techniques have been developed to study respiration and photosynthesis *in vivo*[3]. In green microalgae, they can easily be characterized in terms of O_2_ consumption/production rate of cell suspensions using an aqueous phase Clark-type polarographic electrode. Determination of the apparent maximal activities (MA) of the cytochromial and alternative pathways is enabled by the use of specific inhibitors, i.e. cyanide and substituted hydroxamic acids, respectively [4],[5]. Monitoring chlorophyll fluorescence is also a particularly suitable method for studying the functional properties of the photosynthetic apparatus [6]. In this field, pulse-amplitude modulated (PAM) fluorimetry is the tool of choice, since it enables to monitor chlorophyll fluorescence without any interference of the actinic light applied to induce the biological response [7]. This technology gives access to several important parameters characterizing photosynthesis, notably the quantum yield of photosystem II (ΦPSII) and the non-photochemical quenching of chlorophyll fluorescence (NPQ), which is actually made of 3 components: qE (ΔpH-dependent chlorophyll de-excitation mediated by the xanthophyll cycle), qT (transition of light-harvesting complexes from state 1 to 2) and qI (photoinhibition) [8]–[10]. In contrast to higher plants, state transitions have been demonstrated to be very dynamic in green microalgae, so that qT can importantly contribute to the overall NPQ together with qE [11].

**Figure 1:**
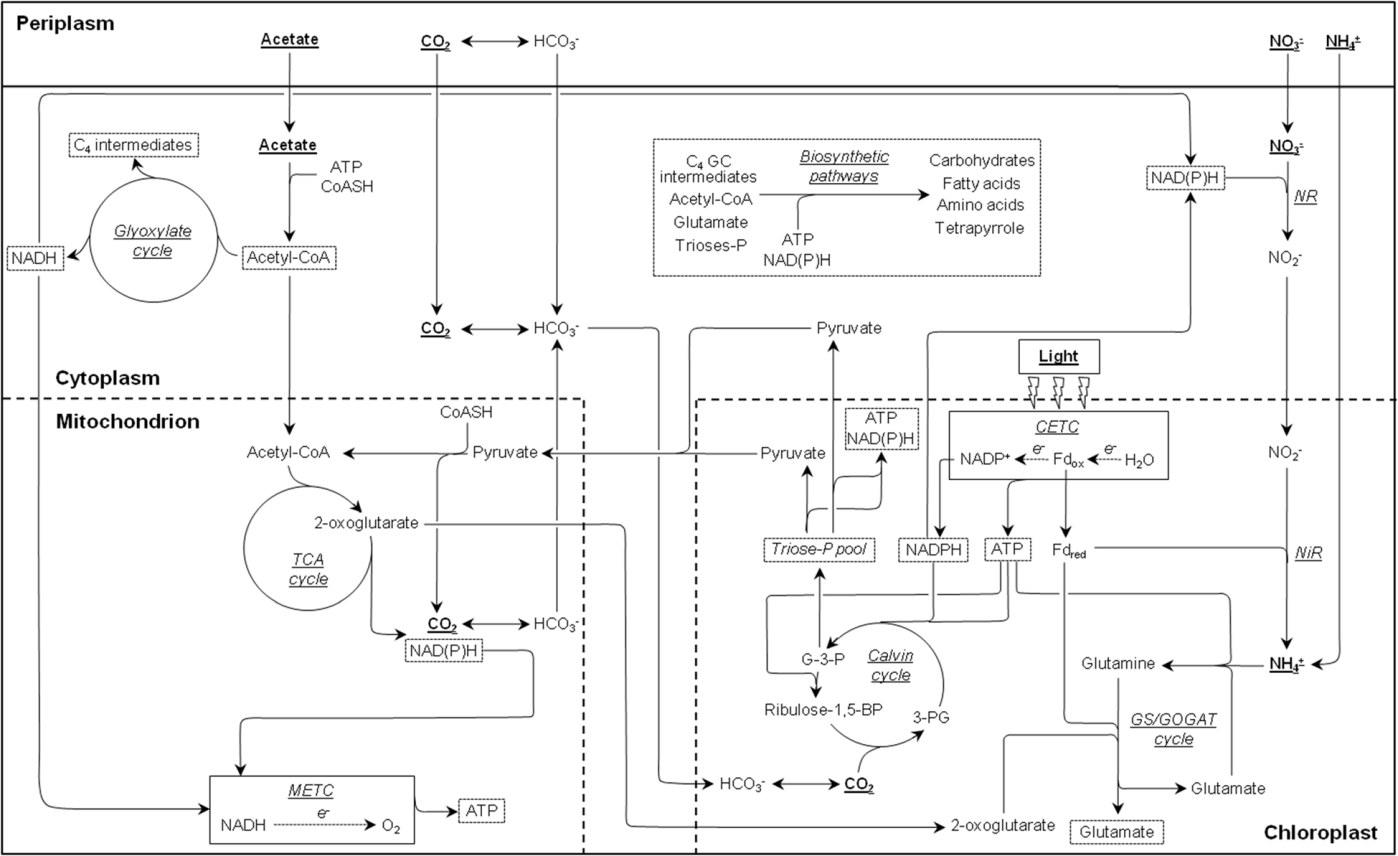
**Assimilatory pathways of light, carbon and inorganic nitrogen in*C. reinhardtii*.** GC, glyoxylate cycle; METC, mitochondrial electron transport chain; CETC, chloroplastic electron transport chain; e^−^, electrons; NR, nitrate reductase; NiR, nitrite reductase; 3-PG, 3-phosphoglycerate; G-3-P, glyceraldehyde-3-phosphate; Fd_red_, reduced ferredoxin; Fd_ox_, oxidized ferredoxin.

The unicellular green alga *Chlamydomonas reinhardtii* is considered as a model to study the metabolism and bioenergetics of photosynthetic organisms [12]. As shown in Figure 1, *C. reinhardtii* is not only able to grow photoautotrophically by using light energy to fix CO_2_ into organic molecules, but can also assimilate acetate as an exogenous organic carbon source under the form of acetyl-CoA through ATP-dependent enzymatic reactions [13]. These features enable cells to grow mixotrophically in the light by harnessing inorganic (CO_2_) and organic (acetate) carbon sources, and even heterotrophically in the dark by oxidizing acetyl-CoA through the glyoxylate and tricarboxylic acid (TCA) cycles to promote the production of reduced cofactors and ATP. The glyoxylate cycle, which bypasses the 2 decarboxylation steps of the TCA cycle, also accounts for the net biomass accumulation because its C_4_ intermediates can be used in biosynthetic pathways [14]. An important feature of CO_2_ fixation in *C. reinhardtii* relies on the carbon concentrating mechanism (CCM), a whole-cell enzymatic machinery enabling to increase CO_2_ availability in the local environment of Rubisco through the dehydration of accumulated bicarbonate to counterbalance the weak catalytic activity of the enzyme and limit its oxygenase activity under low CO_2_ conditions [14],[15]. The CCM consists of several isoforms of carbonic anhydrases (CA) catalyzing the interconversion of CO_2_ and bicarbonate in different sub-cellular compartments but also of diverse inorganic carbon membrane transporters [16],[17].

*C. reinhardtii* can assimilate nitrate and ammonium as inorganic nitrogen sources, but ammonium is preferred to nitrate when they are present together in the medium, consistently with the lower energy cost of ammonium assimilation [18],[19]. Nitrate assimilation first requires its reduction into ammonium, which can then be incorporated within organic molecules under the form of glutamate through the GS/GOGAT cycle in the chloroplast (Figure 1) [20],[21]. Reduced cofactors and ATP being necessary for inorganic nitrogen assimilation mainly originate from photophosphorylation under photoautotrophic culture conditions, but also from the glyoxylate and TCA cycles coupled to oxidative phosphorylation in mixotrophically-grown cells [22],[23].

As illustrated in Figure 1 and demonstrated by recent bioinformatics- and literature-based models of the *C. reinhardtii* metabolic network, the assimilatory pathways of light, carbon and inorganic nitrogen are tightly interconnected through complex exchanges of metabolites, energy and reducing power which are strictly regulated in response to environmental changes to maintain cellular homeostasis [24]–[28]. These features strongly suggest that bioenergetics is likely to be influenced by the cumulative effect of different factors and even by interactions between some of them. However, to our knowledge, most reported studies have only focused on the qualitative or semi-quantitative influence of one or a few environmental factor(s) on respiration and/or photosynthesis while other factors were kept constant.

Design of experiments (DOE) coupled to multiple regression are powerful statistical tools to model the dependence of a physical, chemical or biological process to different intrinsic or extrinsic factors with a limited number of experiments [29]. They are commonly used in diverse applied research fields, in particular for the screening of culture conditions aiming to heighten the production of biomass and/or molecules of interest (metabolites, high-added value compounds, pharmaceutical recombinant proteins, etc.) by diverse organisms [30]–[33]. The methodology has successfully been applied to different microalgae to optimize culture medium for heterotrophic growth, starch and lipid production, CO_2_ fixation as well as metabolite extraction for bio-industrial purposes [34]–[38].

In the present work, DOE coupled to standard least squares multiple regression have been used to model the dependence of several respiratory and photosynthetic responses upon the concomitant modulation of light intensity and acetate, CO_2_, nitrate and ammonium concentrations in the culture medium of *C. reinhardtii*. Bioenergetic responses of interest have been defined as the dark cellular respiration (CR) and the apparent maximal activities of the cytochromial (MA_CYT_) and alternative (MA_ALT_) respiratory pathways, as well as the quantum yield of photosystem II in saturating light (ΦPSII_800_), the gross O_2_ evolution (P_800_, apparent photosynthetic rate) and the non-photochemical quenching of chlorophyll fluorescence (NPQ_800_) measured under a light intensity of 800 μmol_photons_.m^−2^.s^−1^, which is sufficient to saturate photosynthesis but not to induce photoinhibition [39]. The main goals of this study were to determine which environmental factor(s) induce(s) most of the response variability (in other words, which factor(s) mostly account(s) for bioenergetic plasticity) and to characterize quantitatively the influence of these major explanatory factors. Such goals have been achieved through a 2 step approach consisting of a 1st-round of multiple regression aiming to detect the factor(s) of interest, which were then selected for a 2nd-round to generate predictive models enabling to simulate the mathematical profile of their influence. We discuss the results with regards to data reported in literature and we propose biological hypotheses attempting to rationalize the present observations and to provide new promising tracks for future in-depth investigations of the molecular mechanisms underlying bioenergetic plasticity in photosynthetic organisms.

## Results

### Design of experiments

**Figure 2:**
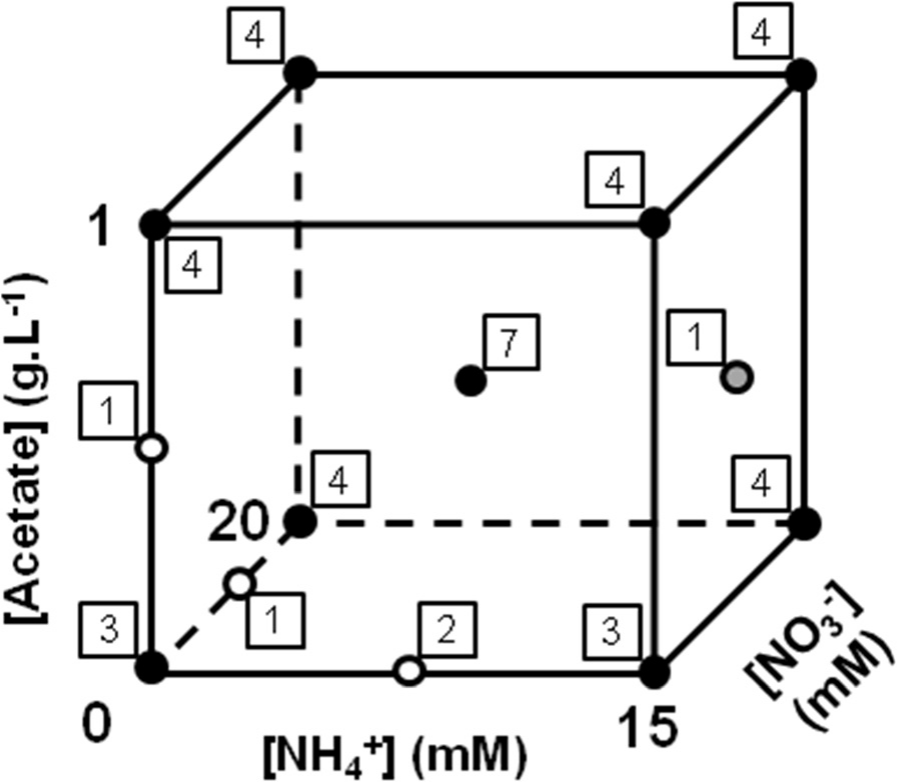
**Factor dispatching within the design space for acetate, ammonium and nitrate concentrations.** Each point is an item of the DOE and its occurrence is indicated by a surrounding framed number. Black points correspond to the fractional factorial basis of the design with additional center points. Grey and white points are extra-points characterizing the central composite and box-Behnken types of design, respectively.

A DOE assuming first- and second-order effects was constructed on the basis of the features summarized in Table 1 to define a limited number of combinations of values for the different environmental factors which altogether were sufficient to cover the whole design space. JMP calculated that the DOE had to contain at least 27 combinations for an unbiased subsequent modeling, and we decided to raise this number up to 42 (+50%) to ensure more confidence toward the analyses. The resulting DOE is a 2 level fractional factorial design with additional center points (i.e. combinations for which all values are equal to the center of the working range) with some extra-points typically characterizing the central composite and box-Behnken types of designs [see Additional file 1 for a complete list of all DOE items] [33]. A 3 dimensional representative example of factor dispatching within the design space is illustrated in Figure 2 for acetate, ammonium and nitrate concentrations.

**Table 1:** Characteristics of the factors in DOE

**Factor**	**Type**	**Unit**	***x***_***min***_**/*M***_***1***_	***x***_***max***_**/*M***_***2***_
Light intensity	Continuous	μmol_photons_.m^−2^.s^−1^	0	200
Acetate concentration	Continuous	g.L^−1^	0	1
CO_2_ concentration	Ordinal (2 M)	%	0.035	1.5
Nitrate concentration	Continuous	mM	0	20
Ammonium concentration	Continuous	mM	0	15

M, modality; *x*_*min*_ and *x*_*max*_, minimal and maximal values of the working range (for continuous variables); M_1_ and M_2_, 2 modalities of CO_2_ concentration.

Pearson's correlation coefficients (*r*) were calculated to verify that altogether the different DOE items were uniformly covering all directions of the design space. These analyses demonstrated that the factors did not relevantly correlate to each other (|*r*| ≤ 0.0382, data not shown). However, while also considering second-order effects, a much more important correlation was detected in a small number of cases (Table 2). Remarkably, an absolute correlation of approximately 0.70 was systematically observed between each single factor and its combination with CO_2_ concentration. This feature does not reflect a sub-optimal experimental design (which would generate a lack of information in some regions of the design space), but is rather an artificial bias attributable to the definition of CO_2_ concentration as an ordinal factor. Due to this particularity, contrarily to the other second-order interactions (which all imply 2 continuous factors), the combination between CO_2_ concentration and another factor cannot be expressed as a continuous product term, but rather as a 2 component term with a specific modality for CO_2_ concentration and a specific numeric value for the other interacting factor. In such a situation, calculating the correlation between a single factor and its interaction with CO_2_ concentration implies to correlate this factor with itself. The observation of an absolute correlation coefficient inferior to 1 is only attributable to the definition in DOE of a different number of points for the 2 CO_2_ modalities for each value of the other interacting factor. As shown in Table 2, an important correlation (|*r*| = 0.5940) could also be noticed between squared acetate and nitrate concentrations, which indicates a lack of test points in a particular region of the design space. Such a collinearity can generate a bias in modeling (due to leverage effects of isolated points) only if these squared concentrations are implied together in the statistical analyses (as discussed below).

**Table 2:** **Significantly correlated effects and associated absolute Pearson's correlation coefficients (|** ***r|*)**

**Factor 1**	**Factor 2**	***|r|***
[Acetate]*[CO_2_]	[Acetate]	0.7135
Light*[CO_2_]	Light	0.6968
[NH_4_^+^]*[CO_2_]	[NH_4_^+^]	0.7092
[NO_3_^−^]*[CO_2_]	[NO_3_^−^]	0.7135
[Acetate]*[Acetate]	[NO_3_^−^]*[NO_3_^−^]	0.5940

The symbol "*" is used to represent second-order effects (quadratic or interactions) of individual factors.

### Measurements of the responses

**Figure 3:**
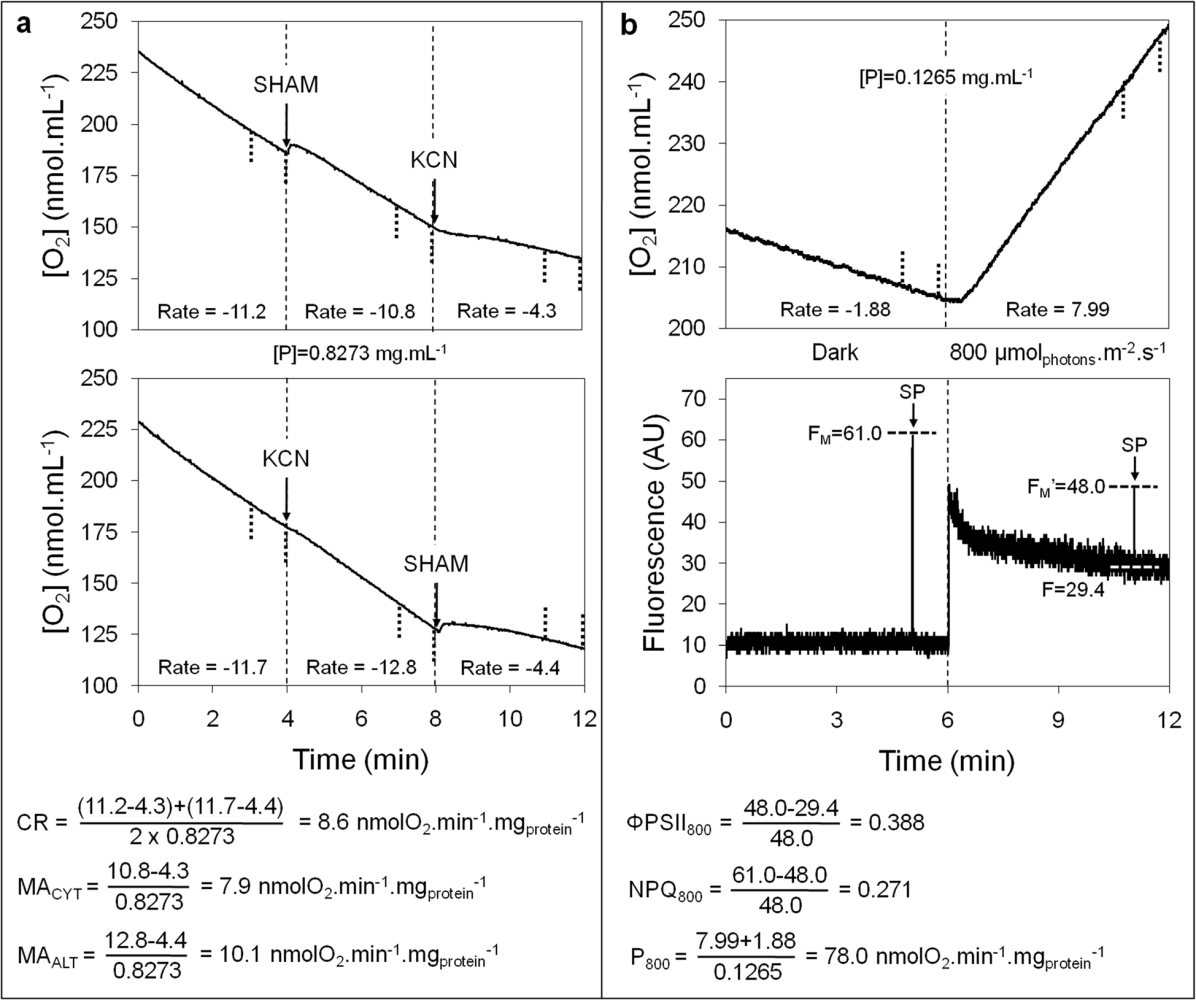
**Oxymetric (a) and chlorophyll fluorescence (b) traces and related calculations for a DOE center point.** Displayed measurements are those of item 4 in Additional file 1. The small vertical bold dotted lines define the zones which were used for rate determinations. AU, arbitrary unit. [P], protein concentration of algal suspension. SP, saturating light pulse.

Bioenergetic responses were measured for the 42 combinations of factors defined in DOE and are summarized in Additional file 1. Measurements were not replicated because a global compensation of the individual experimental errors was expected in subsequent regression processes. Figure 3 presents typical oxymetric and chlorophyll fluorescence traces obtained for one of the center points (DOE item 4) and explains the calculations of respiratory (panel a) and photosynthetic (panel b) responses. It has to be noticed that CR and MA_CYT_ could not be measured in a few cases (DOE items 14 and 28 for CR, 26 for MA_CYT_) but that the amount of experimental data (40 and 41, respectively) remains nevertheless largely sufficient for an unbiased subsequent modeling. As emphasized by the high relative standard deviations of the data (RSD = 29% for ΦPSII_800_ and ≥50% for the other responses, data not shown), every response is distributed within a wide range of values, which provides a highly favorable experimental background for regression processes.

### Selection of the major explanatory factor(s) (1st-round of modeling)

The 1st-round of modeling was performed using the available set of experimental data to detect the factor(s) accounting for most of the response variability. First- and second-order effects were assumed, i.e. it was hypothesized that the responses could depend on each factor linearly or quadratically (for continuous factors) but also on the interaction between 2 factors. This feature rose up to 19 the number of effects which had actually to be involved in the modeling processes, making necessary to get a previous selection to avoid any possible underestimation of important contributions. For this purpose, stepwise regressions were undertaken to define the subset of effects which would provide altogether the smallest AICc (corrected Akaike information criterion) in subsequent modeling. The selected effects are listed in Table 3 with the corresponding AICc, clearly showing that the latter is always dramatically lower than the AICc including all 19 initial effects.

**Table 3:** Summary of the 1st-round of modeling

**CR**				**MA**_**CYT**_				**MA**_**ALT**_			
AICc_ALL_ = 263.56 AICc_MOD_ = 203.75				AICc_ALL_ = 285.34 AICc_MOD_ = 228.73				AICc_ALL_ = 264.77 AICc_MOD_ = 219.39			
R^2^ = 0.83 R^2^ adjusted = 0.81				R^2^ = 0.70 R^2^ adjusted = 0.64				R^2^ = 0.74 R^2^ adjusted = 0.68			
RMSE_F_ = 2.7 (37% of the average scale)				RMSE_F_ = 3.4 (45% of the average scale)				RMSE_F_ = 2.7 (40% of the average scale)			
Whole-model ANOVA: *p* < 0.0001*				Whole-model ANOVA: *p* < 0.0001*				Whole-model ANOVA: *p* < 0.0001*			
Lack-of-fit: *p* = 0.4295				Lack-of-fit: *p* = 0.9282				Lack-of-fit: *p* = 0.6110			
	***Factor***	***β***	***p-value***		***Factor***	***β***	***p-value***		***Factor***	***β***	***p-value***
**1**	**[Acetate]**	**0.660**	<0.0001*	**1**	**[Acetate]**	**0.608**	<0.0001*	**1**	**[NH**_**4**_^**+**^**]**	**−0.465**	<0.0001*
**2**	**[NH**_**4**_^**+**^**]**	**−0.471**	<0.0001*	**2**	**Light**	**0.405**	0.0002*	**2**	**Light**	**0.391**	0.0001*
**3**	**Light**	**0.367**	<0.0001*	**3**	**[NH**_**4**_^**+**^**]**	**−0.355**	0.0007*	**3**	**[Acetate]**	**0.348**	0.0004*
**4**	[Acetate]*[NH_**4**_^**+**^**]**	**−0.292**	0.0002*	4	[NH_4_^+^]*[NO_3_^−^]	0.155	0.1125	**4**	[Acetate]*[Acetate]	**−0.292**	0.0026*
5	[Acetate]*Light	0.160	0.0297*	5	[Acetate]*Light	0.136	0.1635	**5**	[Acetate]*[NH_**4**_^**+**^**]**	**−0.252**	0.0078*
				6	[NO_3_^−^]	−0.095	0.3243	6	[NH_4_^+^]*[NO_3_^−^]	0.248	0.0090*
								7	[NH_4_^+^]*Light	−0.171	0.0637
								8	[NO_3_^−^]	0.100	0.2671
**ΦPSII**_**800**_				**NPQ**_**800**_				**P**_**800**_			
AICc_ALL_ = −53.30 AICc_MOD_ = −110.93				AICc_ALL_ = −25.05 AICc_MOD_ = −78.73				AICc_ALL_ = 422.01 AICc_MOD_ = 373.30			
R^2^ = 0.80 R^2^ adjusted = 0.76				R^2^ = 0.52 R^2^ adjusted = 0.44				R^2^ = 0.75 R^2^ adjusted = 0.68			
RMSE_F_ = 0.056 (28% of the average scale)				RMSE_F_ = 0.081 (40% of the average scale)				RMSE_F_ = 16.4 (37% of the average scale)			
Whole-model ANOVA: *p* < 0.0001*				Whole-model ANOVA: *p* < 0.0001*				Whole-model ANOVA: *p* < 0.0001*			
Lack-of-fit: *p* = 0.1462				Lack-of-fit: *p* = 0.2226				Lack-of-fit: *p* = 0.0874			
	***Factor***	***β***	***p-value***		***Factor***	***β***	***p-value***		***Factor***	***β***	***p-value***
**1**	**Light**	**0.804**	<0.0001*	**1**	**[NO**_**3**_^**−**^]*[NO_**3**_^**−**^**]**	**−0.418**	0.0011*	**1**	**Light**	**0.579**	<0.0001*
2	Light*[NH_4_^+^]	−0.243	0.0029*	**2**	[Acetate]*Light	**−0.360**	0.0041*	**2**	**[NO**_**3**_^**−**^]*[NO_**3**_^**−**^**]**	**−0.755**	0.0002*
3	[Acetate]*[Acetate]	−0.225	0.0062*	**3**	**[Acetate]**	**−0.354**	0.0046*	**3**	[Acetate]*[Acetate]	**0.554**	0.0039*
4	[Acetate]	0.161	0.0424*	4	[NH_4_^+^]	−0.202	0.0928	**4**	**[NO**_**3**_^**−**^**]**	**0.273**	0.0043*
5	[CO_2_]	−0.153	0.0534	5	Light	−0.126	0.2919	5	[NH_4_^+^]*[NO_3_^−^]	−0.251	0.0083*
6	[NH_4_^+^]	0.044	0.5675	6	[NO_3_^−^]	−0.114	0.3388	6	[Acetate]*Light	−0.216	0.0214*
								7	[CO_2_]	0.152	0.0991
								8	[NH_4_^+^]	−0.129	0.1555
								9	[Acetate]	−0.048	0.5948

AICc_ALL_ and AICc_MOD_ correspond to hypothetical models which would comprise every 19 initial effects and the 1st-round models which only contain the stepwise-selected effects, respectively. RMSE_F_ standardized in terms of percentage of the average scale (i.e. the difference between the mean and minimal experimental response values) is displayed to facilitate error comparison among models. Numbers ranging from 1 to 9 classify the different effects by increasing order of individual *p*-value, and β-weights are also provided. Statistically significant *p*-values (*p* ≤ 0.05) are surrounded by *. Effects which are highlighted in bold were considered as major explanatory ones and selected for the 2nd-round of modeling.

Modeling was performed through standard least squares multiple regression with the stepwise-selected effects to establish a predictive mathematical equation [provided in Additional file 2] associating a theoretical response with each of the 42 experimental values [exhaustive list in Additional file 1]. In equations, CO_2_ concentration (which is a factor for ΦPSII_800_ and P_800_) is present under the form of an additional extension term due to its definition as an ordinal factor: this extension is equal to zero for 0.035% CO_2_ and different from zero for 1.5% CO_2_ [Additional file 2]. As summarized in Table 3, R^2^, R^2^ adjusted (which introduces a trade-off for the number of effects to enable proper comparisons among models) and fitting root-mean-square error (RMSE_F_) were calculated to evaluate the goodness of fit, and whole-model and lack-of-fit ANOVA tests were performed to assess the statistical significance of the models. RMSE_F_ was also standardized in terms of percentage of the average scale of the response (i.e. the difference between the mean and the minimal experimental values) in order to facilitate comparisons of regression error among models. Remarkably, the statistical insignificance of lack-of-fit tests (*p* > 0.05 in every model) suggests that no important effect remained unselected through the stepwise regression processes. The values of R^2^, R^2^ adjusted (≥0.70 and 0.64, respectively, except in case of NPQ_800_ for which R^2^ = 0.52 and R^2^ adjusted = 0.44) and RMSE_F_ (≤45% of the average scale) as well as the *p*-values of the whole-model ANOVA tests (*p* < 0.0001 in every model) tend to indicate that altogether the effects selected through stepwise regression account for an important part of the response variability. The relative contribution of the different effects to the models was further investigated by calculating ANOVA tests and β-weights (i.e. regression coefficients which would result from modeling with previous standardization of all variables to a mean of 0 and a variance of 1) for individual effects (Table 3). The absolute value of the latter parameter participates (together with the *p*-value of the ANOVA test) to characterize the extent to which the effect contributes to the model, and its sign assesses whether the effect exerts a positive or negative influence on the response. As shown in Table 3 (in which the effects are classified by increasing order of *p*-value), high absolute β-weights are always associated with small *p*-values. In most cases, β-weights and individual ANOVA tests lead to establish the same order of importance for the different effects, with the exception of P_800_ for which light intensity exhibits a smaller *p*-value than squared nitrate concentration (0.0001 versus 0.0002) but not a higher absolute β-weight (0.579 versus −0.755); however, the 2 parameters similarly point out that these effects are major explanatory ones.

In case of P_800_, 2 second-order effects which had been shown to correlate to each other with *|r|* = 0.5940, i.e. squared acetate and nitrate concentrations (Table 2), were selected through the stepwise regression process. These effects exhibit high absolute β-weights and small *p*-values (β = 0.554/*p* = 0.0039 and β = −0.755/*p* = 0.0002, respectively) comparatively to most of the other effects of the model (Table 3), suggesting that the regression could have been affected by a lack of experimental data in some regions of the design space. However, the observation of an insignificant lack-of-fit ANOVA test (*p* = 0.0874) and of similar R^2^ adjusted and standardized RMSE_F_ values (R^2^ adjusted = 0.68; RMSE_F_ = 37% of the average scale) than those of the other models (except NPQ_800_ which exhibits a lower R^2^ adjusted; see Table 3) tend to indicate that no bias was introduced due to this correlation.

As shown in Table 3, one or several effect(s) which do not exert any statistically significant influence (*p* ≤ 0.05 cutoff) can be found within the models, and considerable differences can even be observed among the relative contribution of the statistically significant effects as indicated by the important heterogeneity of their *p*-values (<0.0001 to 0.0424) and absolute β-weights (0.160 to 0.804). These features suggest that some effect(s) within the models do not substantially influence the responses despite their selection through the stepwise regression processes. Consequently, to avoid overfitting and select the effects which altogether are sufficient to explain most of the response variability, a trial-and-error method consisting of several steps of multiple regression was used: 1st-round effects were removed successively by descending order of *p*-value (from 9 to 1, see Table 3) and modeling was tested with the remaining effects at each step of the process. The remaining effects were considered as major explanatory ones when the removal of the less important of them (i.e. the one exhibiting the highest *p*-value) led to the observation of a significant lack-of-fit ANOVA test (*p* ≤ 0.05) and/or a R^2^ coefficient lower than 0.60 (except in case of NPQ_800_ for which R^2^ = 0.52 in the 1st-round model) (data not shown). As expected, the selected effects (bold characters in Table 3) had been shown to exhibit small *p*-values (*p* < 0.01) and high absolute β-weights (≥0.252) in the 1st-round models comparatively to the unselected effects, which confirms their particularly important contribution.

As emphasized in Table 3 (bold characters), maximum 3 different environmental factors over 5 (with squared effects and second-order interactions in some cases) appear to be sufficient to explain an important part of the response variability. Acetate concentration and light intensity are major explanatory factors for every response except for ΦPSII_800_, for which light intensity is the only one. In addition, ammonium and nitrate concentrations seem to influence to a large extent respiratory (CR, MA_CYT_, MA_ALT_) and other photosynthetic (NPQ_800_, P_800_) responses, respectively, unlike CO_2_ concentration which does not appear to be an important effector of bioenergetic plasticity.

### Mathematical simulation of the influence of the major explanatory factor(s) (2nd-round of modeling)

The 2nd-round of multiple regression was performed with the effects selected through the 1st-round (bold characters in Table 3) in order to build a simplified model for each response. A few unselected first-order effects were also included in the modeling processes for NPQ_800_ (light intensity and nitrate concentration) and P_800_ (acetate concentration) because of the selection of second-order effects containing these factors (Table 3). This process led to establish a simplified mathematical expression (Equations 1 to 6) associating a new predicted response with each of the 42 experimental values:

(1) $$\mathrm{CR}=6.67+9.01\;\left[\mathrm{Acetate}\right]+0.0259\;\mathrm{Light}-0.428\;\left[N{H_{4}}^{+}\right]-0.566\;\left(\left[\mathrm{Acetate}\right]-0.463\right)\;\left(\left[N{H_{4}}^{+}\right]-7.69\right)$$

(2) $$MA_{\mathrm{CYT}}=5.81+7.58\;\left[\mathrm{Acetate}\right]+0.0264\;\mathrm{Light}-0.283\;\left[N{H_{4}}^{+}\right]$$

(3) $$MA_{\mathrm{ALT}}=11.1+3.64\;\left[\mathrm{Acetate}\right]+0.0210\;\mathrm{Light}-0.318\;\left[N{H_{4}}^{+}\right]-13.5\;{\left(\left[\mathrm{Acetate}\right]-0.488\right)}^{2}-0.383\;\left(\left[\mathrm{Acetate}\right]-0.488\right)\;\left(\left[N{H_{4}}^{+}\right]-7.32\right)$$

(4) $$\mathrm{\Phi PSI}I_{800}=0.308+0.00103\;\mathrm{Light}$$

(5) $$\begin{array}{l} \mathrm{NP}Q_{800}=0.334-0.0877\;\left[\mathrm{Acetate}\right]-0.000146\;\mathrm{Light}-0.00146\;\left[N{O_{3}}^{-}\right]\\ -0.00109\;{\left(\left[N{O_{3}}^{-}\right]-9.76\right)}^{2}-0.000918\;\left(\left[\mathrm{Acetate}\right]-0.488\right)\;\left(\mathrm{Light}-107\right) \end{array}$$

(6) $$P_{800}=42.3-3.18\;\left[\mathrm{Acetate}\right]+0.185\;\mathrm{Light}+0.879\;\left[N{O_{3}}^{-}\right]+183\;{\left(\left[\mathrm{Acetate}\right]-0.488\right)}^{2}-0.608\;{\left(\left[N{O_{3}}^{-}\right]-9.76\right)}^{2}$$

**Figure 4:**
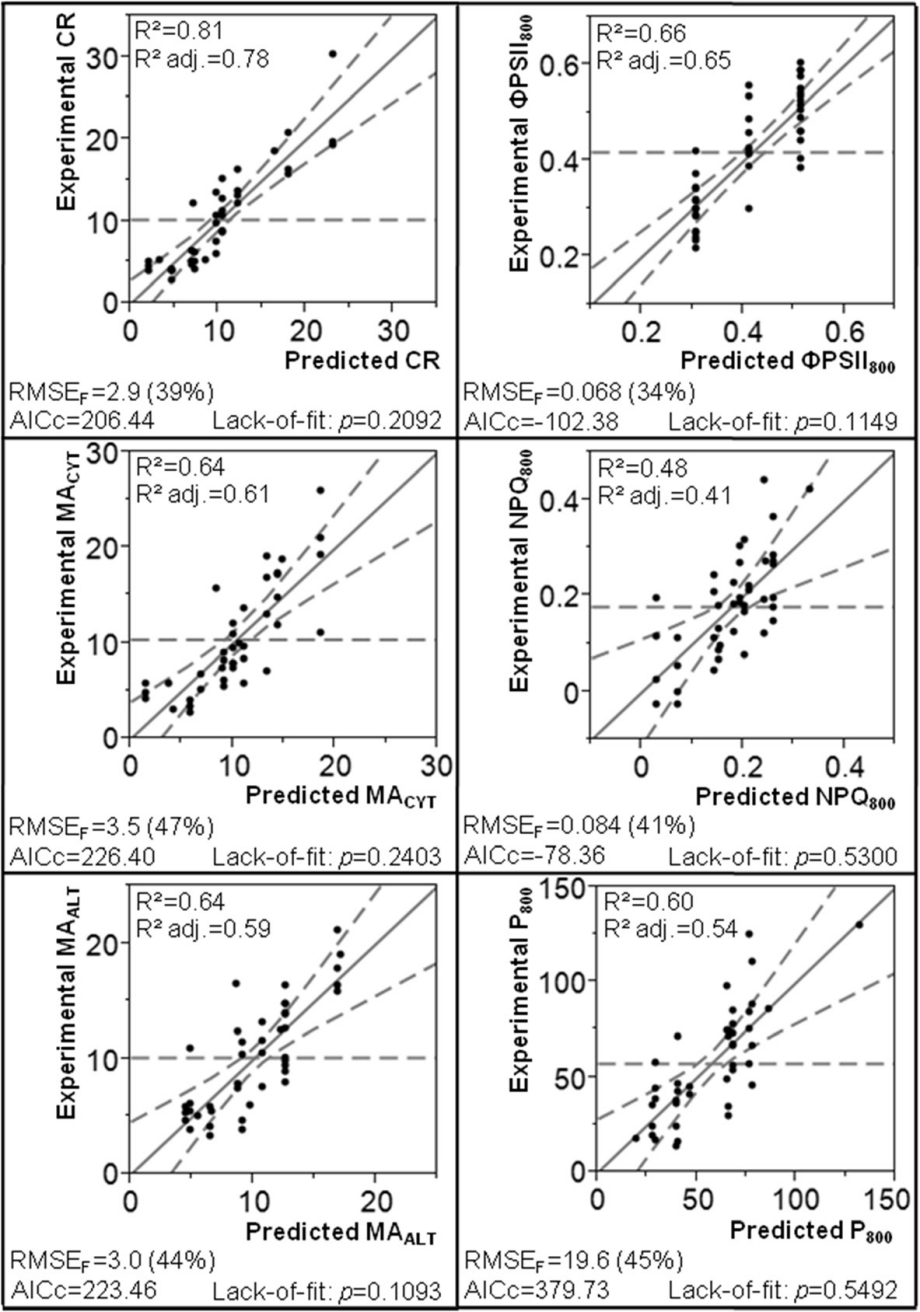
**Experimental values (*y*) as a function of predicted responses (*ŷ*) for the 2nd-round of modeling.** The diagonal full straight line and the curved dotted lines indicate a perfect match of the model (*y = ŷ*) and the 95%-confidence intervals of the whole-model ANOVA test, respectively. The horizontal dotted straight line represents the mean of the response as experimentally observed. The value indicated in parentheses is the RMSE_F_ standardized in terms of percentage of the average scale of the response (i.e. the difference between the mean and the minimal experimental values). R^2^ adj., R^2^ adjusted.

An exhaustive list of the 2nd-round predicted responses is provided in Additional file 1, which also summarizes the 1st-round predicted responses obtained prior to the restriction of the models to the major explanatory factor(s). AICc, R^2^, R^2^ adjusted, RMSE_F_, β-weights and ANOVA tests for whole-model, individual effects and lack-of-fit were calculated and are summarized in Figure 4 and Additional file 3. Comparatively to the 1st-round models, the observations that R^2^ and R^2^ adjusted decreased by maximum 0.15 and 0.14 units, respectively, and that AICc and RMSE_F_ did not increase by more than 8.6 units and 8% of the response average scale, respectively, tend to confirm that the eliminated effects did not substantially account for the response variability. As expected, β-weights and *p*-values of the individual ANOVA tests are similar to those of the 1st-round [see Additional file 3], so that the relative contribution of the essential effects described in Table 3 seems to be globally conserved.

**Figure 5:**
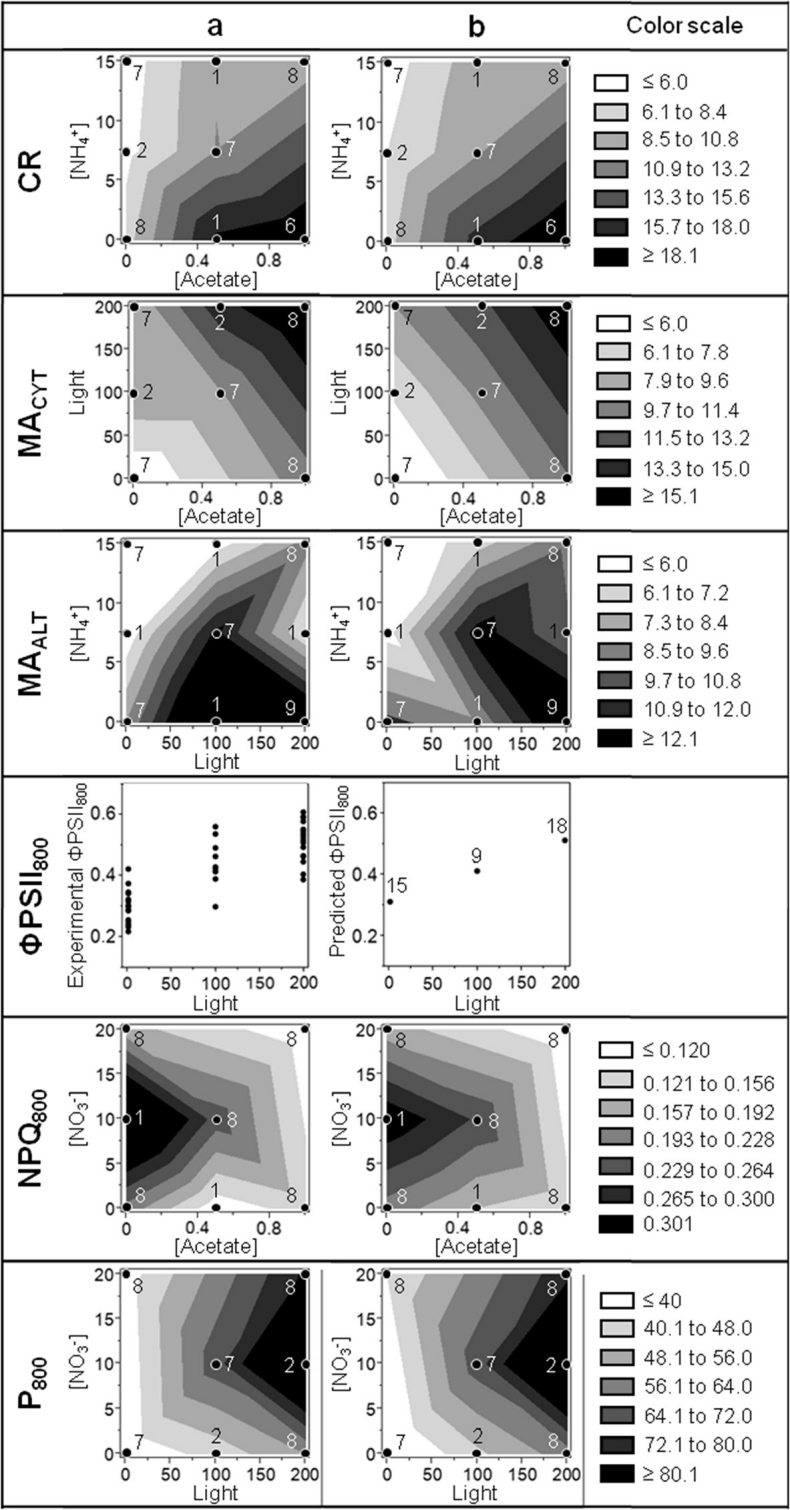
**Comparative profiles of experimental (a) and predicted (b) responses for the 2nd-round of modeling.** Graphs were drawn using JMP on the basis of the 2 explanative factors with the highest individual ANOVA *p*-value. Data points are positioned on the graphs and their occurrence is indicated by surrounding numbers. For ΦPSII_800_, line plots are presented instead of contour plots because light intensity is the only factor of the model.

In Figure 4, experimental values (*y*) are plotted as a function of predicted responses (*ŷ*) calculated from Equations 1 to 6. The *y = ŷ* straight line (corresponding to a perfect match of the model) and the 95%-confidence intervals of the whole-model ANOVA tests are also represented for visual evaluation of goodness of fit and statistical significance, respectively (the more the confidence intervals are close to the *y = ŷ* straight line, the more the statistical significance of the model is important). R^2^, R^2^ adjusted and RMSE_F_ of the different responses (quantifying the goodness of fit) are shown to be ≥0.60, ≥0.54 and ≤47% of the response average scale, respectively, except in case of NPQ_800_ for which R^2^ = 0.48 and R^2^ adjusted = 0.41. ANOVA test *p*-values (characterizing the statistical significance of the models) appear to be inferior or equal to 0.0002 [see Additional file 3]. In Figure 5, contour plots representing the evolution of the experimental (panel a) and predicted (panel b) responses as a function of the 2 factors exhibiting the smallest individual effect *p*-values [see Additional file 3] are compared and reveal closely related global profiles. For ΦPSII_800_, contour plots were replaced by line plots since this response was modeled with only 1 factor (light intensity) in the 2nd-round. These different observations tend to confirm that altogether the effects included in the 2nd-round of modeling are sufficient to explain an important part of the response variability.

**Figure 6:**
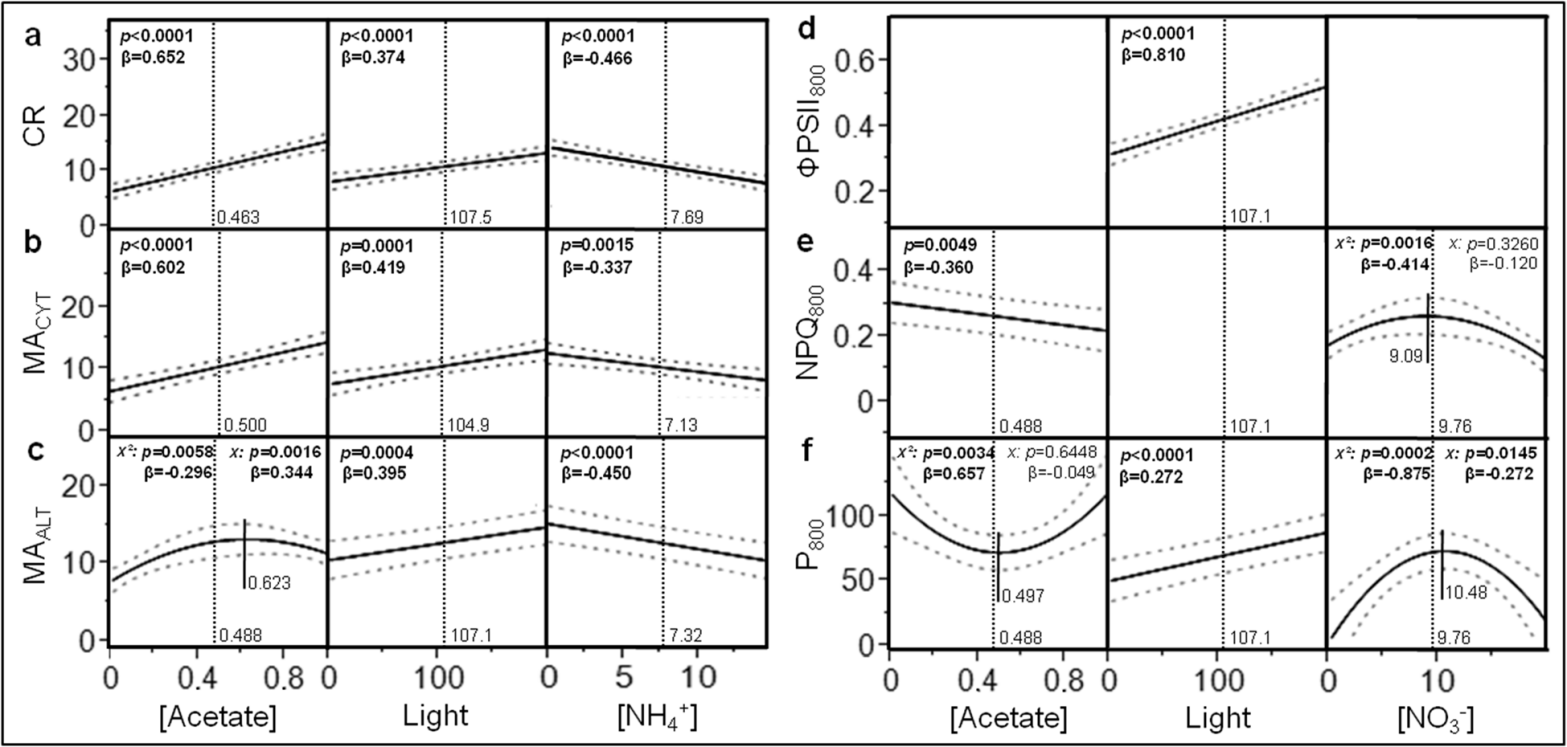
**Simulation of the influence of each major explanatory factor as predicted by the 2nd-round models.** Panels **a** to **c** are for respiratory responses (CR, MA_CYT_ and MA_ALT_, respectively). Panels **d** to **f** are for photosynthetic responses (ΦPSII_800_, NPQ_800_ and P_800_, respectively). The black vertical dotted lines indicate the arithmetic DOE mean, and the small black vertical full straight lines the factor value for which the response is maximal or minimal in case of quadratic profile. The grey curved dotted lines are the 95%-confidence intervals of the simulation. β-weights and individual ANOVA *p*-values [see also Additional file 3] are displayed and highlighted in bold characters while statistically significant (*p* ≤ 0.05). For quadratic profiles, these parameters are provided for the second- and first-order parameter estimates (symbolized by *x*^*2*^ and *x*, respectively).

Figure 6 presents a graphical simulation of the influence of each factor as predicted by the 2nd-round models with the corresponding β-weight(s) and individual effect ANOVA test *p*-value(s) [see also Additional file 3]. The mathematical profile of each factor was established by applying a specific value (i.e. the arithmetic mean of all DOE items) to the other factors of the model within Equations 1 to 6 to generate a new single-unknown predictive equation. In case of quadratic profile, the factor value for which the response is maximal or minimal (for concave and convex shapes, respectively) is displayed and the β-weights and *p*-values are provided for the first- and second-order parameter estimates. The profile of light intensity is not presented for NPQ_800_ since this factor does not relevantly account for response variability *per se* (β = −0.120/*p* = 0.3280); its presence in the model is exclusively due to a second-order interaction with acetate concentration as detailed below. As expected, in case of linear dependence, increasing and decreasing profiles are associated with positive and negative β-weights, respectively; for quadratic relationships, the type of dependence (convex or concave) is pointed out by the sign of the β-weight of the second-order effect (characterizing the second degree coefficient of the polynomial simulation equation), which is positive for convex profiles and negative for concave ones.

As illustrated in Figure 6, CR linearly depends on acetate concentration (β = 0.652), light intensity (β = 0.374) and ammonium concentration (β = −0.466) with *p* < 0.0001 (Figure 6a). MA_CYT_ exhibits a similar dependence upon these factors with the following parameters: β = 0.602/*p* < 0.0001 for acetate concentration, β = 0.419/*p* = 0.0001 for light intensity and β = −0.337/*p* = 0.0015 for ammonium concentration (Figure 6b). MA_ALT_ is linearly modulated by light intensity (β = 0.395/*p* = 0.0004) and ammonium concentration (β = −0.450/*p* < 0.0001), and is also quadratically influenced by acetate concentration (β = −0.296/*p* = 0.0058 and β = 0.344/*p* = 0.0016 for the second- and first-order parameter estimates, respectively; maximum for 0.623 g.L^−1^) (Figure 6c). ΦPSII_800_ and P_800_ linearly increase with light intensity (β = 0.810 and 0.572, respectively) with *p* < 0.0001. In addition P_800_ also quadratically depends on nitrate (β = −0.875/*p* = 0.0002 and β = 0.272/*p* = 0.0145 for the second- and first-order parameter estimates, respectively; maximum for 10.5 mM) and acetate (β *=* 0.657/*p* = 0.0034 and β = −0.049/*p* = 0.6448 for the second- and first-order parameter estimates, respectively; minimum for 0.497 g.L^−1^) (Figure 6d and f). NPQ_800_ is quadratically modulated by nitrate concentration (β = −0.414/*p* = 0.0016 and β = −0.120/*p* = 0.3260 for the second- and first-order parameter estimates, respectively; maximum for 9.1 mM), and also linearly decreases with acetate concentration (β = −0.360/*p* = 0.0049) (Figure 6e).

**Figure 7:**
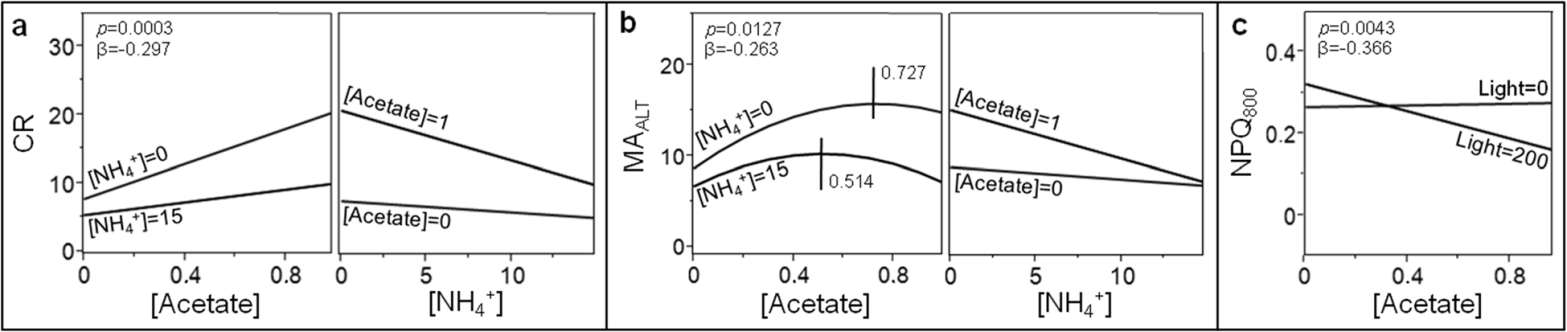
**Second-order interactions characterizing the 2nd-round models.** Panels **a**, **b** and **c** are for CR, MA_ALT_ and NPQ_800_, respectively. The β-weight and individual ANOVA *p*-value are provided for each interaction. In panel **b**, the small black vertical full straight lines with surrounding numbers indicate the optimal acetate concentrations.

In most cases, mathematical profiles (Figure 6) are not influenced by the other factors of the model since modifying their value only induces a translation of the graphs along the ordinate axis without alteration of their general shape. However, as revealed by the occurrence of product terms within Equations 1 to 6 (second-order interactions), a mutual influence between the individual effects of acetate and ammonium concentrations can be observed in case of CR (β = −0.297/*p* = 0.0003) and MA_ALT_ (β = −0.263/*p* = 0.0127), as well as a strong influence of light intensity on the individual effect of acetate concentration in case of NPQ_800_ (β = −0.366/*p* = 0.0043). The negative sign of β-weights indicates that heightening the value of one of the interacting factors leads to a decrease of the slope (i.e. the first degree coefficient of the simulation equation) characterizing the profile of the other factor. In Figure 7, the influence of each interacting factor is independently simulated for 2 different values of the other one (i.e. the minimal and maximal values of the DOE range; see Table 1) by the same procedure as described for Figure 6 (the third, non interacting factor is kept equal to the arithmetic DOE mean). No graph is presented for light intensity in case of NPQ_800_ since this factor poorly contributes to the model (β = −0.120/*p* = 0.3280) whatever the acetate concentration is. Remarkably, for CR and MA_ALT_, the effects of acetate and ammonium concentrations appear to be weakened and strengthened, respectively, by increasing the value of the other factor (Figure 7a and b). Concerning MA_ALT_, rising ammonium concentration also generates a displacement of the optimal acetate concentration towards smaller values (maximum for 0.727 and 0.514 g.L^−1^ in case of 0 and 15 mM ammonium, respectively). For NPQ_800_, a relevant effect of acetate concentration is only observed upon high irradiance (Figure 7c).

### Validation of the 2nd-round models: cross-validation and experimental validation on new random combinations

In parallel to the main goals of the present study (i.e. to explain response variability and to characterize the influence of the major explanatory factors), we wondered whether the 2nd-round models could also be used as tools to predict the responses associated with any combination of factors within the range of DOE (Table 1).

In order to investigate the predictive ability of the 2nd-round models, experimental data were divided into *k* = 5 subsets (defined in Additional file 1) and *k*-fold cross-validation was undertaken. Each of the 5 test sets was compared to the corresponding training model (built from all data except those of the test set) in terms of cross-validation mean absolute error (MAE_CV_) and root-mean-square error (RMSE_CV_). Table 4 presents for each response the average MAE_CV_ and RMSE_CV_ summarizing the deviation of the 5 test data sets to their respective training model, as well as the average R^2^, R^2^ adjusted, MAE_F_ (fitting mean absolute error) and RMSE_F_ summarizing the analytical error of the 5 training models (for an exhaustive description of cross-validation results, see Additional file 4). The average scales of the different responses are also provided as a reference to assess the extent of the deviations. As shown in Table 4, the R^2^, R^2^ adjusted, MAE_F_ and RMSE_F_ of the training models are identical or very similar to those of the 2nd-round models (which are also presented in Table 4 to facilitate result overview). Remarkably, the MAE_CV_ and RMSE_CV_ characterizing the deviation of the test data sets to their respective training model do not exceed the analytical error of more than 37% for MAE_CV_ and 21% for RMSE_CV_, and these proportions are reduced to 29% and 12%, respectively, if P_800_ is not taken into account.

**Table 4:** Summary of cross-validation and experimental validation of the 2nd-round models

	**2nd-round models**				***K*-fold cross-validation (average values for*k*** **= 5)**						**Experimental validation sets**		**Average scale**
					**Training models**				**Test sets**				
	**R**^**2**^	**R**^**2**^**adj.**	**MAE**_**F**_	**RMSE**_**F**_	**R**^**2**^	**R**^**2**^**adj.**	**MAE**_**F**_	**RMSE**_**F**_	**MAE**_**CV**_	**RMSE**_**CV**_	**MAE**_**EV**_	**RMSE**_**EV**_	
**CR**	0.81	0.78	2.2	2.9	0.81	0.79	2.2	2.8	2.6	3.1	1.9	2.2	7.4
**MA**_**CYT**_	0.64	0.61	2.7	3.5	0.66	0.62	2.6	3.5	3.2	3.9	1.7	2.1	7.5
**MA**_**ALT**_	0.64	0.59	2.2	3.0	0.65	0.59	2.1	3.0	2.7	3.2	1.8	1.9	6.8
**ΦPSII**_**800**_	0.66	0.65	0.053	0.068	0.65	0.64	0.053	0.068	0.054	0.067	0.068	0.078	0.201
**NPQ**_**800**_	0.48	0.41	0.062	0.084	0.50	0.41	0.061	0.083	0.076	0.093	0.039	0.044	0.205
**P**_**800**_	0.60	0.54	13.6	19.6	0.60	0.54	13.4	19.6	18.4	23.7	17.3	23.4	43.9

Cross-validation was performed by the *k*-fold method with *k* = 5; the R^2^, R^2^ adjusted, MAE and RMSE presented for the training models (“F”) and the test data sets (“CV”) are the average values compiling the 5 iterations. For each response, the average scale (i.e. the difference between the mean and minimal experimental values of the DOE study) is also provided as a reference to assess the importance of the deviations. R^2^ adj., R^2^ adjusted.

**Figure 8:**
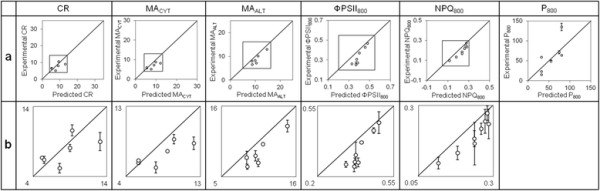
**Mean experimental values as a function of predicted responses for random combinations in Table**5**.** Mean experimental values (ȳ) result from 3 independent sets of cultures and measurements. Predicted responses (*ŷ*) were calculated from Equations 1 to 6 (2nd-round models). The diagonal full straight line represents a perfect match of the model (*y = ŷ*). In panel **a**, axis scaling is identical to that of Figure 4 to facilitate visual comparison. The framed zone in panel a is enlarged in panel **b**, in which scaling is adapted to data points and standard deviations are drawn except in case of they are comprised within the limits of the markers. For P_800_, standard deviations are presented in panel a since it was not necessary to adapt scaling.

In addition to cross-validation, the predictive ability of the 2nd-round models was also evaluated by measuring the bioenergetic responses for 6 new randomly-generated combinations of factors (different from any DOE item) with 3 experimental replicates each (independent cultures and functional measurements) (Table 5). Every algal culture was undertaken with 1.5% CO_2_ for technical convenience since CO_2_ concentration was not included in the 2nd-round models. Because of the quite large standard deviations related to NPQ_800_ measurements (RSD = 19.7% on average), 2 more combinations were tested for photosynthetic responses (total number = 8) in order to increase confidence toward the general tendency of data. The predicted responses associated with the random combinations were calculated from Equations 1 to 6, as summarized in Additional file 4. The deviation of the experimental validation data sets (containing 6 or 8 items) to the 2nd-round models was assessed in terms of experimental validation mean absolute error (MAE_EV_) and root-mean-square error (RMSE_EV_) (Table 4); this deviation is also illustrated in Figure 8, in which the experimental values are plotted as a function of the predicted ones. As shown in Table 4, the MAE_EV_ and RMSE_EV_ of the experimental validation data are inferior to the analytical error of the 2nd-round models for CR, MA_CYT_, MA_ALT_ and NPQ_800_; for ΦPSII_800_ and P_800_, they exceed the analytical error of only 28 and 27% for MAE_EV_ and 15 and 19% for RMSE_EV_, respectively. As clearly evidenced while comparing Figure 4 and Figure 8a, it has to be noticed that the responses measured for the new random combinations do not cover the full range observed in the DOE study (except for P_800_). This feature could be attributable to the random choice of the factor values for the 6 or 8 validation points without consideration of the responses predicted by the 2nd-round models.

**Table 5:** Randomly-generated combinations of factors and associated mean experimental responses used for experimental validation

	**[Ac.]**	**Light**	**[NH**_**4**_^**+**^**]**	**[NO**_**3**_^**−**^**]**	**[CO**_**2**_**]**	**CR**	**MA**_**CYT**_	**MA**_**ALT**_	**ΦPSII**_**800**_	**NPQ**_**800**_	**P**_**800**_
**1**	0.58	61	10.0	7.0	1.5	8.1 ± 0.5	7.3 ± 0.2	10.1 ± 0.2	0.273 ± 0.019	0.218 ± 0.020	50.3 ± 1.7
**2**	0.32	58	15.0	3.0	1.5	6.7 ± 0.3	5.8 ± 0.3	6.6 ± 0.4	0.250 ± 0.025	0.183 ± 0.028	58.9 ± 2.4
**3**	0.21	90	15.0	9.0	1.5	6.4 ± 0.5	6.1 ± 0.2	8.0 ± 1.0	0.369 ± 0.005	0.235 ± 0.052	64.0 ± 0.4
**4**	0.75	65	12.0	5.0	1.5	10.6 ± 0.8	8.8 ± 0.6	8.3 ± 0.8	0.307 ± 0.064	0.166 ± 0.075	48.8 ± 1.6
**5**	0.35	144	0.0	10.0	1.5	9.0 ± 1.3	8.3 ± 0.8	12.9 ± 0.9	0.428 ± 0.013	0.244 ± 0.042	134.9 ± 9.1
**6**	0.25	75	7.5	10.0	1.5	5.2 ± 0.7	5.2 ± 0.7	7.2 ± 0.6	0.270 ± 0.022	0.273 ± 0.025	71.3 ± 3.9
**7**	0.82	167	0.0	0.0	1.5				0.467 ± 0.054	0.097 ± 0.021	15.2 ± 3.2
**8**	1.00	21	15.0	0.0	1.5				0.260 ± 0.015	0.133 ± 0.024	23.2 ± 1.9

Data result from 3 independent sets of cultures and measurements. [Ac.], acetate concentration.

Altogether, these different validation results indicate that the 2nd-round models can be used as tools to predict the responses associated with any combination of factors, with an inherent average deviation being quantified by the MAE_F_ and RMSE_F_ characterizing the analytical error of the models. As deduced from Table 4, this deviation is comprised between 26 and 36% of the response average scale for MAE_F_ and 34 and 47% for RMSE_F_.

## Discussion

In the present work, DOE coupled to standard least squares multiple regression have been used to model the dependence of different respiratory (CR, MA_CYT_, MA_ALT_) and photosynthetic (P_800_, ΦPSII_800_, NPQ_800_) responses upon the concomitant modulation of light, carbon and inorganic nitrogen sources in the culture medium of *C. reinhardtii*. This methodology was applied to characterize the extent to which the different environmental factors contribute to bioenergetic plasticity (through a 1st-round of modeling) as well as the mathematical profile of their influence for those accounting for most of the response variability (through a 2nd-round of modeling). Altogether, these analyses provide an overview of the bioenergetic adaptations resulting from global changes in culture conditions. This type of sequential statistical approach, which is commonly undertaken for the optimization of industrial production yields and the design and analysis of “-omics” experiments, had never been used to characterize the bioenergetic plasticity of photosynthetic cells. The individual influence exerted by one or a few environmental factor(s) (maintaining the others constant) on the cellular bioenergetics and metabolism had extensively been studied independently, but little information was available concerning their cumulative effect and their relative contribution to bioenergetic plasticity in a context in which they vary concomitantly in the medium.

The present analyses demonstrate that maximum 3 environmental factors over 5 are sufficient to explain most of the response variability (Table 3) and remarkably evidence squared effects and second-order interactions in some cases (Figure 6; Figure 7). As shown in Figure 4, comparatively to the other responses, lower R^2^ and R^2^ adjusted characterize the 2nd-round models obtained for NPQ_800_ (this difference was also noticed in the 1st-round models; see Table 3). Such discrepancies could (at least partly) be attributable to the higher experimental error inherent to NPQ_800_ measurements (see in Table 4 and Figure 8b the large standard deviations among the 3 experimental replicates of the random combinations tested for model validation: on average, RSD = 19.7% for NPQ_800_ and ≤9.0% for the other responses).

In order to check whether the 2nd-round models could also be used to predict the responses associated with any combination of factors within the range of DOE (Table 1), *k*-fold cross-validation and experimental validation tests on new random combinations have been undertaken. The similarity between the deviation of the validation points (quantified using MAE_CV_/MAE_EV_ and RMSE_CV_/RMSE_EV_) and the analytical error of the training or 2nd-round models (for cross-validation and experimental validation, respectively) tend to confirm the predictive ability of the 2nd-round models (Table 4). It must nevertheless be emphasized that deviations of 26 to 36% of the response average scale (in terms of MAE_F_) or 34 to 47% (in terms of RMSE_F_) corresponding to the analytical error of the 2nd-round models are inherent to the predictions.

In literature, O_2_ evolution commonly appears to be normalized in terms of chlorophyll concentration. However, in the present analyses, it was rather chosen to use protein concentration because of the high dependence of *C. reinhardtii* pigment content upon culture conditions, particularly light and acetate [40]–[42]. Our unconventional normalization strategy could therefore generate apparent discrepancies between previously reported studies and the present results in some cases.

In the following sections, the authors will attempt to propose literature-based hypotheses addressing the possible biological implications of their observations. They insist on emphasizing that these hypotheses must not be considered as firm assertions, but rather aim to provide tracks for future in-depth molecular investigations.

### Light stimulates CO_2_ fixation through the Calvin cycle and provides mitochondrial respiration with oxidizable substrates

The gross photosynthetic O_2_ evolution and the quantum yield of photosystem II of *C. reinhardtii* cells adapted to moderate light intensities (0–200 μmol_photons_.m^−2^.s^−1^) have been measured under 800 μmol_photons_.m^−2^.s^−1^. Under such a so-called “saturating” intensity, the electron transport rate (ETR) is not limited by light availability but rather by the capacity of downstream metabolic pathways that consume photo-generated reductant and ATP (such as the Calvin cycle). In these conditions, the gross O_2_ evolution (which is partly mediated by the rate of water photolysis) can primarily be considered as representative of the capacity of these pathways, even if numerous studies indicate that complex photosynthesis-associated O_2_-consuming processes (particularly PTOX chlororespiration and Mehler reaction) can also importantly contribute in some circumstances to modulate this response in *C. reinhardtii*[43].

The present analyses indicate that light intensity exerts a positive linear influence on ΦPSII_800_ and P_800_ (Figure 6d and f). Accordingly, the maximal gross O_2_ evolution was previously reported to be doubled in *C. reinhardtii* cells grown under illumination of 400 μmol_photons_.m^−2^.s^−1^ comparatively to a lower illumination of 50 μmol_photons_.m^−2^.s^−1^[40]. These observations could (at least partly) be attributable to the well-known stimulation of the expression and activity of Calvin cycle enzymes by light [44], in good agreement with the higher CO_2_ fixation rates observed upon increasing illumination in *C. reinhardtii*[45]. This improvement of CO_2_ fixation by light was reported to be correlated to higher cellular metabolite content, respiratory O_2_ consumption and TCA cycle-mediated CO_2_ production, in line with the linear stimulation of CR, MA_CYT_ and MA_ALT_ by light which could also be detected here (Figure 6a to c). For MA_CYT_ and MA_ALT_, the term “apparent” is used because measurements were carried out on entire cells but not on isolated mitochondria. The availability of respiratory substrates could therefore not be directly controlled, so that the measured maximal activities could have been underestimated comparatively to the actual capacities if the intracellular reductant concentration was insufficient to saturate the mitochondrial electron transport chain in the presence of KCN or salicylhydroxamic acid (SHAM).

In apparent contradiction with these considerations, the present analyses did not retain CO_2_ concentration as a major explanatory factor of bioenergetic plasticity (Table 3). Such an absence of influence had already been highlighted for the maximal gross O_2_ evolution in a previous study, in which the sum between the net O_2_ evolution monitored under 600 μmol_photons_.m^−2^.s^−1^ and the dark respiration measured before illumination was shown to be similar in low and high CO_2_-grown *C. reinhardtii* cells [46]. These observations could be explained by the existence of a low CO_2_-inducible CCM in *C. reinhardtii*, by which a high CO_2_ availability for Rubisco is maintained in low CO_2_ condition. Several transcriptomic analyses demonstrated that adaptation to different CO_2_ concentrations mainly occurs through the regulation of the genetic expression of CCM components but not Calvin cycle enzymes in *C. reinhardtii*[47]–[49]. Moreover, transferring *C. reinhardtii* cells from high to low CO_2_ external concentration was shown to result in a transient decrease of the amount of the small and large Rubisco subunits before returning (within the time period required to induce CCM) to the levels characterizing high CO_2_-grown cells [50]. Altogether, these different observations and the present ones tend to indicate that adaptation to low CO_2_ environment in *C. reinhardtii* principally occurs through CCM induction but not Calvin cycle regulation.

### Acetate down-regulates the capacity of the Calvin cycle and promotes its own uptake and storage to counteract the osmotic stress associated with high extracellular acetate concentrations

The present analyses indicate that P_800_ depends on acetate concentration following a quadratic convex profile with a minimal value for 0.497 g.L^−1^ (Figure 6f). In a previous study, the net O_2_ evolution measured under 600 μmol_photons_.m^−2^.s^−1^ was shown to decrease with acetate concentrations ranging from 0 to 1.75 g.L^−1^, but rates had been normalized in terms of chlorophyll concentration and cultures conducted under the same saturating light intensity than that of measurements (600 μmol_photons_.m^−2^.s^−1^) [51].

In *C. reinhardtii* and *Chlorogonium elongatum* (a closely related unicellular green alga), acetate is known to repress the expression of the genes encoding the small and large Rubisco subunits (*rbcS* and *rbcL*, respectively), thereby lowering the capacity for CO_2_ fixation through the Calvin cycle [51]–[53]. In this context, carbon originating from acetate can substitute for up to half the photoautotrophically-generated biomass content [51],[54], and light-driven photosynthetic reactions importantly contribute to provide reductant and ATP for biosynthetic acetate assimilation (as shown in *Chlamydomonas mundane*) [55]. In heterotrophically-grown *C. reinhardtii* cells, acetate storage as starch is also known to be promoted through the improvement of the expression and activity of enzymes of the glyoxylate cycle (as isocitrate lyase, ICL) and gluconeogenesis [56],[57]. In parallel to its influence on carbon metabolism, acetate inhibits *C. reinhardtii* heterotrophic growth beyond 0.4 g.L^−1^ in the medium (“substrate inhibition”) [58]. From this concentration, the osmotic potential reaches a critical value beyond which active transport processes are impaired and energy requirements for cellular maintenance are considerably heightened. Interestingly, for P_800_, the present analyses point out a “concentration of inflexion” (0.5 g.L^−1^ approximately) which is very close to the critical substrate inhibition concentration of 0.4 g.L^−1^ (Figure 6f). This observation tends to indicate that P_800_ could be influenced by 2 independent acetate-responsive metabolic processes consuming photo-generated reductant and ATP: the Calvin cycle (repressed while increasing acetate concentration due to Rubisco down-regulation) and the biosynthetic assimilation of acetate (stimulated while increasing acetate concentration, especially beyond 0.5 g.L^−1^, to promote acetate uptake and storage in order to attenuate the osmotic stress).

### Acetate stimulates mitochondrial respiration by heightening the intracellular reductant content and the capacity of the cytochrome pathway

The present analyses indicate that acetate concentration exerts a positive linear influence on CR and MA_CYT_ (Figure 6a and b). Accordingly, when grown in an acetate-containing medium, *C. reinhardtii* cells were previously shown to exhibit a twice-enhanced respiratory rate (partly due to the improvement of the intracellular reductant content in mixotrophic condition) [59] as well as increased transcript levels for diverse components of oxidative phosphorylation, suggesting a higher capacity of the cytochrome pathway [60]. In parallel, MA_ALT_ depends on acetate concentration following a quadratic concave profile with an optimum for 0.623 g.L^−1^. As illustrated in Figure 6c, this response can nevertheless be considered as linearly stimulated up to 0.5 g.L^−1^ acetate without further increase beyond this concentration. This observation tends to indicate that substrate-saturation of the alternative pathway in the presence of KCN could be reached beyond 0.5 g.L^−1^ acetate, which would imply that AOX capacity is not responsive to acetate concentration. Accordingly, enhancement of the capacity of the cytochrome pathway was already suggested to contribute to the acetate-induced improvement of dark respiration without concomitant modification of AOX capacity [61].

### Acetate inhibits NPQ through repression of the LHCSR3-dependent qE component

The present analyses demonstrate that acetate concentration exerts a negative linear influence on NPQ_800_ (Figure 6e), but only in case of high light intensity (Figure 7c). Interestingly, the extent of qE has recently emerged as being dramatically lowered by the presence of acetate in the growth medium, as notably evidenced by Finazzi and co-workers who demonstrated that qT is the major contributor to the global NPQ in mixotrophically-grown *C. reinhardtii* cells [62]. Even if the molecular mechanisms underlying the functional relationship between NPQ and acetate are not yet understood, the present results tend to confirm these findings and indicate that the magnitude of the inhibitory effect of acetate on qE could depend on its external concentration. Recently, qE has been proposed to be mediated by LHCSR3, a light-harvesting complex orthologue which is only expressed upon high irradiance [63],[64]. NPQ plasticity induced in response to changing environmental conditions (such as different acetate concentrations) could therefore be disabled in the dark and low light intensities due to the down-regulation of LHCSR3. These interpretations must be considered with caution due to the impossibility to distinguish the contributions of qE and qT to the global NPQ here.

### Mitochondrial respiration contributes to provide nitrate assimilation with reductant through the acetate-dependent activity of AOX

Ammonium concentration is shown here to exert a negative linear influence on CR, MA_CYT_ and MA_ALT_ (Figure 6a to c). In *Selenastrum minutum* (another green alga), mitochondrial respiration was proposed to play a role in nitrate assimilation by acting as a trigger factor for the TCA cycle. This would in turn promote the production and export of reductant in the cytoplasm and the chloroplast to support nitrate reduction [65]. In *C. reinhardtii*, the enzymatic activity and genetic expression of proteins involved in nitrate assimilation are known to be repressed by ammonium [20],[21]. These regulatory events are responsible for a strict control of inorganic nitrogen uptake and assimilation by ammonium availability and enable to preferentially exploit this reduced N form if nitrate is also present in the medium [18]. Such a primary control of nitrate assimilation by ammonium could rationalize the present observations with regards to the postulated role of mitochondrial respiration in this metabolic process.

For MA_ALT_, ammonium concentration is the factor which explains the highest proportion of response variability (β = −0.450/*p* < 0.0001; Figure 6c). Interestingly, the gene encoding AOX (*Aox1*) is known to be located within a gene cluster which also encodes components of the nitrate assimilatory pathway and is tightly regulated by the nitrogen source [66]; consequently, AOX expression and capacity were shown to be induced by nitrate and repressed by ammonium in a concentration-dependent manner [61]. With regards to the peculiar genetic localization and regulation of *Aox1*, the postulated role of mitochondrial respiration in nitrate assimilation was proposed to be essentially mediated by AOX, as also indicated by a recent comparative proteomic study published by our group [67]. The present results are in good agreement with these findings.

Interestingly, a mutual influence could be detected between the individual effects of acetate and ammonium concentrations for CR and MA_ALT_ (second-order interactions). As illustrated in Figure 7a and b (left panels), the effect of acetate concentration is attenuated by ammonium. For MA_ALT_, there is also a displacement of the optimal acetate concentration toward smaller values with increasing ammonium concentration (0.727 and 0.514 g.L^−1^ for 0 and 15 mM ammonium, respectively). These results are consistent with a negative influence of ammonium concentration on AOX capacity. They also tend to confirm that the involvement of mitochondrial respiration in nitrate assimilation is essentially mediated by AOX since no relevant second-order interaction was retained for MA_CYT_. Reciprocally, as illustrated in Figure 7a and b (right panels), ammonium concentration exerts a relevant influence on CR and MA_ALT_ only upon high acetate concentration (these responses exhibit a basal ammonium-independent value in the absence of acetate). This observation tends to indicate that acetate assimilation could provide the TCA cycle with oxidizable substrates to support the involvement of AOX in nitrate assimilation.

### Nitrate assimilation is retro-inhibited to prevent the deleterious effects of nitrite and ammonium intracellular accumulation

The present analyses demonstrate that nitrate concentration exerts a quadratic concave influence on P_800_ with an optimum for 10.48 mM (Figure 6f). In *C. reinhardtii*, photosynthesis is known to contribute to provide nitrate reduction with electrons (together with mitochondrial respiration as stated beyond) [22],[23],[67], so that the rate of nitrate assimilation can influence P_800_ in the same way as for the Calvin cycle. The effect of nitrate on P_800_ can therefore be thought to result (such as for acetate) from 2 distinct metabolic processes of which the relative importance varies with nitrate concentration: substrate stimulation of reductase activity (predominant from 0 to 10 mM) and retro-inhibition of nitrate reduction by nitrate-derived intracellular ammonium (predominant beyond 10 mM). Such a retro-inhibition could attenuate the production of nitrite and ammonium (despite the higher nitrate availability) and prevent the deleterious effects which would result from their intracellular accumulation (nitric oxide overproduction and buffering disturbance, respectively) [20],[68],[69].

Similarly to P_800_, NPQ_800_ also depends on nitrate concentration following a quadratic concave profile with an optimum for 9.09 mM (Figure 6e). Assuming that the NADPH-to-ATP stoechiometric ratio of nitrate assimilation is superior to the yield of photosynthesis, the reoxidation of photo-generated reductant may not be paralleled with ATP turnover. This feature could result in heightening ΔpH across the thylakoid membrane, which would in turn stimulate high energy state chlorophyll de-excitation (qE) in an extent depending on the rate of nitrate assimilation.

## Conclusions

In the present work, DOE coupled to standard least squares multiple regression have been applied to model the dependence of respiration and photosynthesis upon light, carbon and inorganic nitrogen sources in *C. reinhardtii* through a 2 step approach consisting of 2 successive rounds of modeling. This methodology enabled to demonstrate that maximum 3 environmental factors over 5 account for most of the variability of the different responses (i.e. can induce a relevant bioenergetic plasticity) and also permitted to obtain a mathematical simulation of the influence of the major explanatory factors. Altogether, these results provide an overview of the adaptations of *C. reinhardtii* bioenergetic pathways to changing culture conditions and point out new promising tracks for future more specific investigations. In order to further characterize the molecular adaptations underlying the present functional observations, we have undertaken a DOE-based comparative proteomic analysis (using two dimensional-differential in-gel electrophoresis) coupled to the determination of pigment and lipid composition by chromatography, of which the results are currently being modeled.

## Methods

### Algal cultures

The *cw15* mt^+^ wall-less strain 83 of *C. reinhardtii*[70] was pre-cultured at 25°C in 1 L Erlenmeyer flasks under orbital agitation (120 rpm), moderate light intensity (75 μmol_photons_.m^−2^.s^−1^) and ambient air in 400 mL of a classical pre-culture medium (NaNO_3_ 20 mM, K_2_HPO_4_ 5.4 mM, KH_2_PO_4_ 4.6 mM, MgSO_4_ 1.4 mM, CaCl_2_ 450 μM, oligo-elements (H_3_BO_3_ 180 μM, ZnSO_4_ 75 μM, MnCl_2_ 25 μM, FeSO_4_ 18 μM, CoCl_2_ 6.8 μM, CuSO_4_ 6.3 μM, (NH_4_)_6_Mo_7_O_24_ 890 nM), Tris–HCl 20 mM pH 7.2) (photoautotrophic growth). After 6 days, pre-cultured algae were span down by centrifuging at 1,000 *g* for 3 min and washed once in the targeted culture medium by successive resuspension and centrifugation to eliminate pre-culture medium. Algae were finally resuspended in 75 mL of targeted culture medium, transferred to lab-scale tubular photobioreactors (Multi-Cultivators MC 1000, Photon System Instruments) allowing an accurate control of light intensity (from 0 to 200 μmol_photons_.m^−2^.s^−1^) and cultured for 48 h at 25°C prior to performing functional measurements. Algal cultures were bubbled either with ambient air or with a mixture composed of 98.5% ambient air and 1.5% CO_2_. Culture media invariably contained K_2_HPO_4_ 5.4 mM, KH_2_PO_4_ 4.6 mM, MgSO_4_ 1.4 mM, CaCl_2_ 450 μM, oligo-elements (see above), Tris–HCl 20 mM pH 7.2. Acetic acid (0 to 1 g.L^−1^), NaNO_3_ (0 to 20 mM) and/or NH_4_Cl (0 to 15 mM) could also be added to reach a definite concentration depending on the specificity of each culture medium. Given the high dependence of algal growth rate upon environmental conditions, algal cultures were inoculated at a variable initial cellular density allowing to reach a dry biomass concentration of 250 μg.mL^−1^ (approximately corresponding to an absorbance of 1.1 at 750 nm) after 48 h. To measure the dry biomass concentration, samples of algal cultures were washed twice with milliQ water and dried for 24 h at 75°C.

### Measurements of respiratory responses: CR, MA_CYT_, MA_ALT_

48 h-old algal cultures were concentrated up to a 500 μg.mL^−1^ dry biomass to optimize resolution of functional measurements. For this purpose, algae were span down by centrifuging at 1,000 *g* for 3 min, half of the culture medium was removed and algal pellets were resuspended in the remaining volume. Respiratory parameters were determined by oxygen concentration measurements using Clark electrode and related oxygraph device and software (Hansatech). Measurements were carried out in the dark at 25°C. Oxygen concentration was monitored for 4 min before adding SHAM 2 mM (as a specific inhibitor of the alternative pathway) or KCN 2 mM (as a specific inhibitor of the cytochrome pathway). After 4 more min, KCN 2 mM or SHAM 2 mM was further added, respectively, and oxygen concentration was monitored for 4 min before ending record (total time: 12 min). The experiment was carried out twice by reversing the addition order of inhibitors (KCN + SHAM and SHAM + KCN). Cellular respiration (CR) was considered as the oxygen consumption rate in the absence of inhibitors (mean of the 2 independent measurements). Apparent maximal activities of cytochromial (MA_CYT_) and alternative (MA_ALT_) pathways were assessed as the oxygen consumption rates in the presence of SHAM or KCN alone, respectively. In each case, the weak oxygen consumption rate remaining after adding the 2 inhibitors (residual respiration due to inhibitor-insensitive cellular oxidases) was substracted. Oxygen consumption rates were reported to protein concentration of algal suspension and expressed in nmolO_2_.min^−1^.mg_proteins_^−1^.

### Measurements of photosynthetic responses: ΦPSII_800_, NPQ_800_, P_800_

48 h-old algal cultures were diluted down to 80 μg.mL^−1^ dry biomass (approximately corresponding to 8 μg/mL chlorophylls) to avoid any light screen effect which would otherwise affect functional measurements. For this purpose, a culture sample was centrifuged at 10,000 *g* for 3 minutes to spin down algae and the supernatant, only containing culture medium, was added to an untreated culture sample following a 2:1 volume ratio to generate a 3-fold dilution of algal suspension. Photosynthetic parameters were determined using a PAM fluorimeter coupled to an oxymetric device (Clark electrode and oxygraph) (Hansatech). Measurements were carried out at 25°C in the presence of NaHCO_3_ 10 mM to avoid any experimental bias due to CO_2_ limitation. Chlorophyll fluorescence and oxygen concentration were monitored for 6 min without actinic light and for 6 more min under a saturating actinic light of 800 μmol_photons_.m^−2^.s^−1^ (total time: 12 min). 2 pulses of saturating light (5,000 μmol_photons_.m^−2^.s^−1^ for 2.5 s) were applied after 5 and 11 minutes to close transitorily all photosystem II Q_A_ centers, resulting in a brief fluorescence raise before returning instantaneously to the baseline level. Chlorophyll fluorescence traces were analyzed to determine the value of F (fluorescence baseline under 800 μmol_photons_.m^−2^.s^−1^) as well as F_M_ and F_M_’ (maximum fluorescence values reached during the saturating pulse in the dark and under 800 μmol_photons_.m^−2^.s^−1^, respectively). These values were used to calculate ΦPSII_800_ = (F_M_’-F)/F_M_’) [8] and NPQ_800_ = (F_M_-F_M_’)/F_M_’) [9]. Oxygen concentration traces were employed to calculate P_800_ by adding the dark oxygen consumption rate to the net oxygen release rate monitored under 800 μmol_photons_.m^−2^.s^−1^. Oxygen consumption rate was reported to protein concentration of algal suspension and expressed in nmolO_2_.min^−1^.mg_proteins_^−1^.

### Determination of protein concentration

In case of respiratory and photosynthetic measurements, 1 mL and 6 mL of algal suspension, respectively, were centrifuged at 10,000 *g* for 5 minutes. Algal pellets were invariably resuspended in 1 mL extraction buffer (NaCl 150 mM, EDTA 1 mM, Triton X-100 1%, Tris–HCl 50 mM pH 7.5) and 25 mg polyvinylpolypyrrolidone (PVPP, insoluble in aqueous solution) were further added to complex polyphenols which would otherwise interfere with protein assay. Samples were sonicated at 3 Amp for 30 s on ice (Sonifier Cell Disruptor B-12, Branson) and thoroughly vortexed for 5 min at 4°C. PVPP was span down by centrifuging at 10,000 *g* for 5 min and the supernatant was used for determination of protein concentration (mg.mL^−1^) by a Reagent Compatible/Detergent Compatible protein assay kit (Bio-Rad) derived from the Lowry-Ciocalteu method (for details see the manufacturer’s instructions) [71].

### Statistical analyses related to DOE and multiple regression

All statistical analyses were carried out using the JMP 10 software from SAS Institute. The DOE was constructed using the “custom designer” module with a single continuous response as described in Table 1. First- and second-order effects were considered, i.e. it was assumed that the response could depend on each factor linearly or quadratically (for continuous factors) but also on the interaction between 2 factors. The presence of 7 “center points”, i.e. combinations of factors for which all the values are equal to the center of the working range, was imposed in DOE.

Stepwise regressions were undertaken with minimum AICc as stopping rule and the following features as selection process: forward (progressive selection of the effects) and combine (automatic selection of a first-order effect in case of a second-order one containing the same environmental factor is selected). Comparatively to AIC (the corresponding “uncorrected” Akaike information criterion), AICc introduces a supplemental penalty while increasing the number of factors and experimental data to avoid overfitting. It is believed to exhibit theoretical and experimental advantages in the field of regression-based modeling in comparison with other likelihood-derived statistical parameters such as the Bayesian information criterion (BIC) [72].

Modeling was performed through standard least squares multiple regression to minimize the error mean square between experimental (*y*) and predicted (*ŷ*) responses by adjusting the coefficients of the following type of equation:

(7) $$\hat{y}\;=\;b_{0}+\;\sum \;b_{i}x_{i}+\;\sum \;b_{\mathrm{ij}}x_{i}x_{j}+\sum \;b_{\mathrm{ii}}x_{i}^{2}\;+\;e$$

where *b*_*0*_ is the intercept (constant term), *x*_*i or j*_ the factors, *b*_*i*_, *b*_*ij*_ and *b*_*ii*_ the respective linear, interaction and squared parameter estimates and *e* the residual.

**Figure 9:**
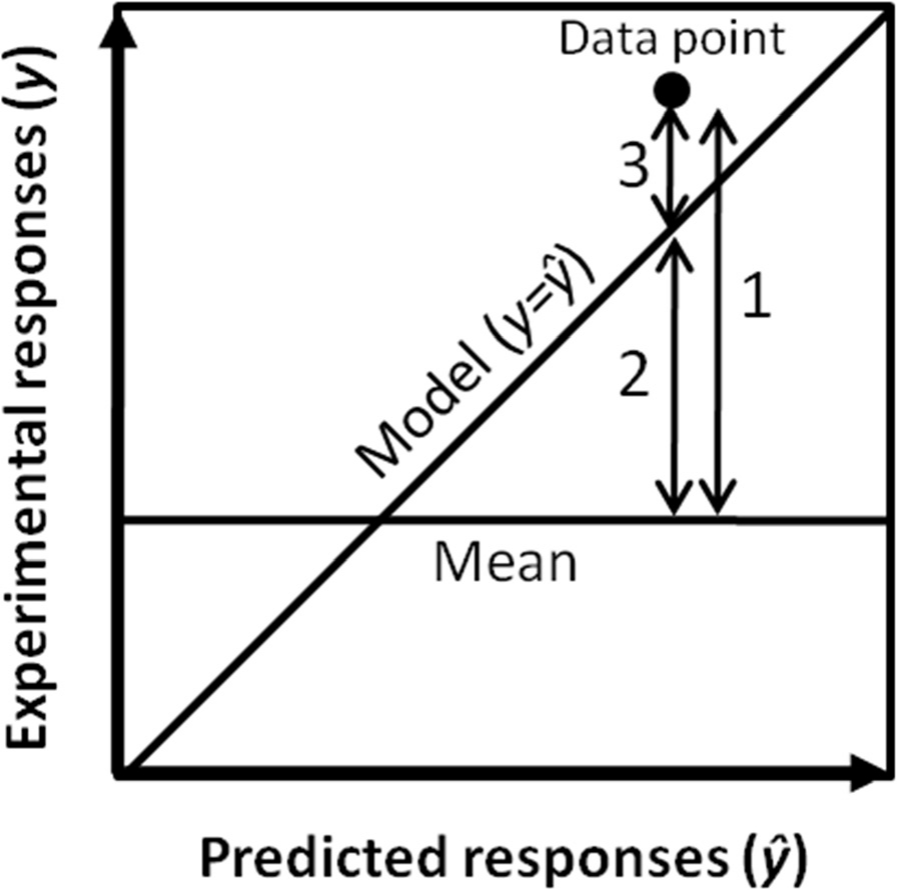
**Distances used for SS determination in the context of R**^**2**^**, R**^**2**^**adjusted, RMSE**_**F**_**and ANOVA test calculations.** Distances 1, 2 and 3 were employed for the assessment of total, model and error SS, respectively.

One-way ANOVA statistical *F*-tests, coefficients of multiple determination (R^2^ and R^2^ adjusted) and fitting root-mean-square error (RMSE_F_) were calculated on the basis of total, model and error sum of squares (SS) resulting from the distances illustrated in Figure 9. ANOVA tests were undertaken on the basis of mean squares (MS) calculated from model and error SS with *k* and *n-k-1* degrees of freedom (DF), respectively, where *k* is the number of factors and *n* the number of experimental values (MS = SS/DF). R^2^ was assessed as the quotient between model and total SS to estimate the proportion of the variability of the response which can be attributed to the model. R^2^ adjusted was calculated as 1-error MS/total MS (with *n-1* DF for determination of total MS) to introduce a trade-off for the number of factors, as required to perform proper comparisons of goodness of fit among different models. RMSE_F_ was obtained from error MS and standardized in terms of percentage of the average scale (i.e. the difference between the mean and the minimal values of experimental data).

Whole-model ANOVA tests were applied to characterize the statistical significance of the models. The *F*-ratio was calculated as the quotient between model and error MS and the null hypothesis (i.e. MS equality) was rejected for *p* ≤ 0.05, indicating the variability of the response to be predominantly attributable to one or several first- or second-order effect(s).

Individual effect β-weights and ANOVA tests were calculated to compare the relative contribution of the different effects to the model. β-weights are the regression coefficients which would be obtained if the different variables had been standardized to a mean of 0 and a variance of 1 prior to modeling. The more the absolute value of this parameter is high, the more the weight of the effect within the model is important. Individual effect ANOVA tests characterize the extent to which the regression error would be increased if modeling was performed without the effect. Effect SS was assessed as the difference between the error SS of the model deprived of the effect and the actual error SS of the model, 1 DF being used for subsequent MS determination. The *F*-ratio was calculated as the quotient between effect and error MS and the null hypothesis was rejected for *p* ≤ 0.05, indicating the effect to be statistically significant (the smaller the *p*-value, the stronger the contribution to the model).

Lack-of-fit tests were carried out to estimate the likelihood for the model to lack one or several important effect(s). Pure error SS was assessed as the error SS of a saturated model, and lack-of-fit SS as the difference between error and pure error SS; for detailed explanation, see [73] and the “Regression Reports” webpage of the JMP online support [74]. The *F*-ratio was calculated as the quotient between lack-of-fit and pure error MS and the null hypothesis was rejected for *p* ≤ 0.05, indicating at least one important effect to be missing in the model.

### Model validation

Cross-validation was performed by the *k*-fold method with *k* = 5 using the Statistica 10 software from Statsoft. The deviation of each test data set to its training model was quantified in terms of MAE_CV_ (cross-validation mean absolute error) and RMSE_CV_ (cross-validation root-mean-square error). R^2^, R^2^ adjusted, MAE_F_ (fitting mean absolute error) and RMSE_F_ inherent to the training models were also calculated to assess the goodness of fit. In addition, responses were measured for several randomly-generated combinations of factors (different from any combination included in DOE) and the deviation of these experimental validation data sets to the 2nd-round models was assessed in terms of MAE_EV_ (experimental validation mean absolute error) and RMSE_EV_ (experimental validation root-mean-square error).

### Artwork and graph designs

Artwork was undertaken using the PowerPoint and Picture Manager softwares of the 2007 Microsoft Office suite. Data obtained from functional measurements were processed using the Excel software from the same company to calculate respiratory and photosynthetic responses and display them graphically. Graphs reporting results of multiple regressions and mathematical simulations (including line plots and contour plots) were drawn using the JMP 10 software from SAS Institute.

## Availability of supporting data

The data sets supporting the results of this article are included within the article and its additional files.

## Abbreviations

ΦPSII: Quantum yield of photosystem II

AICc: Corrected Akaike information criterion

AOX: Mitochondrial alternative oxidase

CA: Carbonic anhydrase

CCM: Carbon-concentrating mechanism

CR: Cellular respiration

DOE: Design of experiments

ETR: Electron transport rate

MA: Apparent maximal activity

MAE_CV_: Cross-validation mean absolute error

MAE_EV_: Experimental validation mean absolute error

MAE_F_: Fitting mean absolute error

MS: Mean square

NPQ: Non-photochemical quenching of chlorophyll fluorescence

P: Gross O_2_ evolution

PAM: Pulse-amplitude modulated

RMSE_CV_: Cross-validation root-mean-square error

RMSE_EV_: Experimental validation root-mean-square error

RMSE_F_: Fitting root-mean-square error

RSD: Relative standard deviation

SHAM: Salicylhydroxamic acid

SS: Sum of squares

TCA: Tricarboxylic acid

## Competing interests

The authors declare that they have no competing interests.

## Authors’ contributions

GM is the author of the original idea of the work. GM and SG conceived the DOE. SG performed algal cultures and functional measurements according to FF’s expertise, as well as data analyses and modeling with GM’s contribution for statistics. SG wrote the manuscript with helpful advice from FF and GM in regards to biological and technical aspects, respectively. All authors read and approved the final manuscript.

## Additional files

## Supplementary Material

Additional file 1 **Table summarizing DOE as well as experimental and predicted responses.** Predicted responses of the 1st- and 2nd-round of modeling were calculated from Additional file 2 and Equations 1 to 6, respectively. Predicted responses of the 1st-round are those obtained before the restriction of the models to the major explanatory factor(s) (this restriction was subsequently applied through the 2nd-round). Data subsets defined for *k*-fold cross-validation are also provided. Click here for file

Additional file 2 **Mathematical equations resulting from the 1st-round of modeling.** These equations were obtained prior to the restriction of the models to the major explanatory factor(s) (as done in the 2nd-round of modeling). Due to its definition as an ordinal factor, CO_2_ concentration is present as an extension term which is equal to zero for 0.035% CO_2_ and different from zero for 1.5% CO_2_. Click here for file

Additional file 3 **β-weights, whole-model and individual effect ANOVA tests of the 2nd-round of modeling.** Numbers ranging from 1 to 5 classify the different effects by increasing order of individual *p*-value. *P*-values which are surrounded by * are considered as statistically significant (*p* ≤ 0.05). Click here for file

Additional file 4 **Supplementary information concerning the validation of the 2nd-round models.** Sheet 1 describes the parameters (R^2^, R^2^ adjusted, MAE_F_/MAE_CV_, RMSE_F_/RMSE_CV_ and whole-model *p*-value) characterizing each of the 5 training models and test sets of *k*-fold cross-validation (set numbering corresponds to that in Additional file 1). Sheet 2 lists the randomly-generated combinations of factors used for experimental validation tests and the associated predicted responses (obtained from Equations 1 to 6). Click here for file

## References

[B1] Mitchell P Coupling of phosphorylation to electron and hydrogen transfer by a chemi-osmotic type of mechanism Nature 1961 191 4784 144 148 10.1038/191144a0 13771349

[B2] Millenaar F Lambers H The alternative oxidase: in vivo regulation and function Plant Biol 2003 5 1 2 15 10.1055/s-2003-37974

[B3] Hunt S Measurements of photosynthesis and respiration in plants Physiol Plant 2003 117 3 314 325 10.1034/j.1399-3054.2003.00055.x 12654031

[B4] Antonini E Brunori M Greenwood C Malmström B Rotilio G The interaction of cyanide with cytochrome oxidase Eur J Biochem 1971 23 2 396 400 10.1111/j.1432-1033.1971.tb01633.x 4333368

[B5] Schonbaum G Bonner WJ Storey B Bahr J Specific inhibition of the cyanide-insensitive respiratory pathway in plant mitochondria by hydroxamic acids Plant Physiol 1971 47 1 124 128 10.1104/pp.47.1.124 5543780 PMC365824

[B6] Baker N Chlorophyll fluorescence: a probe of photosynthesis in vivo Annu Rev Plant Biol 2008 59 89 113 10.1146/annurev.arplant.59.032607.092759 18444897

[B7] Horton P Relations between electron transport and carbon assimilation; simultaneous measurement of chlorophyll fluorescence, transthylakoid pH gradient and O2 evolution in isolated chloroplasts P Roy Soc Lond B Bio 1983 217 1209 405 416 10.1098/rspb.1983.0018

[B8] Genty B Briantais J Baker N The relationship between the quantum yield of photosynthetic electron transport and quenching of chlorophyll fluorescence BBA-Gen Subjects 1989 990 1 87 92 10.1016/S0304-4165(89)80016-9

[B9] Bilger W Björkman O Role of the xanthophyll cycle in photoprotection elucidated by measurements of light-induced absorbance changes, fluorescence and photosynthesis in leaves of Hedera canariensis Photosynth Res 1990 25 3 173 185 10.1007/BF00033159 24420348

[B10] Müller P Li X-P Niyogi K Non-photochemical quenching: a response to excess light energy Plant Physiol 2001 125 4 1558 1566 10.1104/pp.125.4.1558 11299337 PMC1539381

[B11] Allorent G Tukotsu R Roach T Peers G Cardol P Girard-Bascou J Seigneurin-Berny D Petroutsos D Kuntz M Breyton C Franck F Wollman F Niyogi K Krieger-Liszkay A Minagawa J Finazzi G A dual strategy to cope with high light in Chlamydomonas reinhardtii Plant Cell 2013 25 2 545 557 10.1105/tpc.112.108274 23424243 PMC3608777

[B12] Harris E Chlamydomonas as a model organism Annu Rev Plant Physiol Plant Mol Biol 2001 52 1 363 406 10.1146/annurev.arplant.52.1.363 11337403

[B13] Ingram-Smith C Martin S Smith K Acetate kinase: not just a bacterial enzyme Trends Microbiol 2006 14 6 249 253 10.1016/j.tim.2006.04.001 16678422

[B14] Spalding M Stern D The CO2-Concentrating Mechanism and Carbon Assimilation The Chlamydomonas Sourcebook Organellar and Metabolic Processes 2009 2 257 301 10.1016/B978-0-12-370873-1.00016-2

[B15] Meyer M Griffiths H Origins and diversity of eukaryotic CO2-concentrating mechanisms: lessons for the future J Exp Bot 2013 64 3 769 786 10.1093/jxb/ers390 23345319

[B16] Moroney J Ma Y Frey W Fusilier K Pham T Simms T DiMario R Yang J Mukherjee B The carbonic anhydrase isoforms of Chlamydomonas reinhardtii: intracellular location, expression, and physiological roles Photosynth Res 2011 109 1–3 133 149 10.1007/s11120-011-9635-3 21365258

[B17] Spalding M Microalgal carbon-dioxide-concentrating mechanisms: Chlamydomonas inorganic carbon transporters J Exp Bot 2008 59 7 1463 1473 10.1093/jxb/erm128 17597098

[B18] Thacker A Syrett P The assimilation of nitrate and ammonium by Chlamydomonas reinhardi New Phytol 1972 71 3 423 433 10.1111/j.1469-8137.1972.tb01942.x

[B19] Florencio F Vega J Utilization of nitrate, nitrite and ammonium by Chlamydomonas reinhardii Planta 1983 158 4 288 293 10.1007/BF00397329 24264747

[B20] Fernández E Galván A Quesada A Netherlands S Nitrogen Assimilation and its Regulation The Molecular Biology of Chloroplasts and Mitochondria in Chlamydomonas 2004 637 659 10.1007/0-306-48204-5_33

[B21] Fernández E Galván A Nitrate assimilation in Chlamydomonas Eukaryot Cell 2008 7 4 555 559 10.1128/EC.00431-07 18310352 PMC2292633

[B22] Turpin D Effects of inorganic N availability on algal photosynthesis and carbon metabolism J Phycol 1991 27 1 14 20 10.1111/j.0022-3646.1991.00014.x

[B23] Huppe H Turpin D Integration of carbon and nitrogen metabolism in plant and algal cells Annu Rev Plant Physiol Plant Mol Biol 1994 45 1 577 607 10.1146/annurev.pp.45.060194.003045

[B24] Chang R Ghamsari L Manichaikul A Hom E Balaji S Fu W Shen Y Hao T Palsson B Salehi-Ashtiani K Papin J Metabolic network reconstruction of Chlamydomonas offers insight into light-driven algal metabolism Mol Syst Biol 2011 7 1 518 10.1038/msb.2011.52 21811229 PMC3202792

[B25] Boyle N Morgan J Flux balance analysis of primary metabolism in Chlamydomonas reinhardtii BMC Syst Biol 2009 3 1 4 10.1186/1752-0509-3-4 19128495 PMC2628641

[B26] Gomes de Oliveira Dal'Molin C Quek L Palfreyman R Nielsen L AlgaGEM - a genome-scale metabolic reconstruction of algae based on the Chlamydomonas reinhardtii genome BMC Genomics 2011 12 Suppl 4 S5 10.1186/1471-2164-12-S4-S5 PMC3287588 22369158

[B27] Kliphuis A Klok A Martens D Lamers P Janssen M Wijffels R Metabolic modeling of Chlamydomonas reinhardtii: energy requirements for photoautotrophic growth and maintenance J Appl Phycol 2012 24 2 253 266 10.1007/s10811-011-9674-3 22427720 PMC3289792

[B28] May P Wienkoop S Kempa S Usadel B Christian N Rupprecht J Weiss J Recuenco-Munoz L Ebenhöh O Weckwerth W Walther D Metabolomics- and proteomics-assisted annotation and analysis of the draft metabolic network of Chlamydomonas reinhardtii Genetics 2008 179 1 157 166 10.1534/genetics.108.088336 18493048 PMC2390595

[B29] Steinberg D Hunter W Experimental design: review and comment Technometrics 1984 26 2 71 97 10.1080/00401706.1984.10487928

[B30] Datta P Linhardt R Sharfstein S An 'omics approach towards CHO cell engineering Biotechnol Bioeng 2013 110 5 1255 1271 10.1002/bit.24841 23322664

[B31] Alam M Jamal P Nadzir M Bioconversion of palm oil mill effluent for citric acid production: statistical optimization of fermentation media and time by central composite design World J Microbiol Biotechnol 2008 24 7 1177 1185 10.1007/s11274-007-9590-5

[B32] Sathiyamoorthy P Shanmugasundaram S Experimental design for optimization of cyanobacterial biomass production in a low-cost bioreactor Bioresource Technol 1995 53 3 225 229 10.1016/0960-8524(95)00088-V

[B33] Tye H Application of statistical 'design of experiments' methods in drug discovery Drug Discov Today 2004 9 11 485 491 10.1016/S1359-6446(04)03086-7 15149624

[B34] Azma M Mohamed M Mohamad R Rahim R Ariff A Improvement of medium composition for heterotrophic cultivation of green microalgae, Tetraselmis suecica, using response surface methodology Biochem Eng J 2011 53 2 187 195 10.1016/j.bej.2010.10.010

[B35] Huang G-H Chen G Chen F Rapid screening method for lipid production in alga based on Nile red fluorescence Biomass Bioenerg 2009 33 10 1386 1392 10.1016/j.biombioe.2009.05.022

[B36] Dragone G Fernandes B Abreu A Vicente A Teixeira J Nutrient limitation as a strategy for increasing starch accumulation in green microalgae Appl Energ 2011 88 10 3331 3335 10.1016/j.apenergy.2011.03.012

[B37] Anjos M Fernandes B Vicente A Teixeira J Dragone G Optimization of CO2 bio-mitigation by Chlorella vulgaris Bioresource Technol 2013 139 149 154 10.1016/j.biortech.2013.04.032 23648764

[B38] Serive B Kaas R Bérard J-B Pasquet V Picot L Cadoret J-P Selection and optimization of a method for efficient metabolites extraction from microalgae Bioresource Technol 2012 124 311 320 10.1016/j.biortech.2012.07.105 22989659

[B39] Falk S Samuelsson G Recovery of photosynthesis and photosystem II fluorescence in Chlamydomonas reinhardtii after exposure to three levels of high light Physiol Plant 1992 85 1 61 68 10.1111/j.1399-3054.1992.tb05264.x

[B40] Neale P Melis A Algal photosynthetic membrane complexes and the photosynthesis-irradiance curve: a comparison of light adaptation responses in Chlamydomonas reinhardtii (Chlorophyta) J Phycol 1986 22 4 531 538 10.1111/j.1529-8817.1986.tb02497.x

[B41] Falkowski P LaRoche J Acclimation to spectral irradiance in algae J Phycol 1991 27 1 8 14 10.1111/j.0022-3646.1991.00008.x

[B42] Polle J Benemann J Tanaka A Melis A Photosynthetic apparatus organization and function in the wild-type and a chlorophyll b-less mutant of Chlamydomonas reinhardtii: dependence on the carbon source Planta 2000 211 3 335 344 10.1007/s004250000279 10987551

[B43] Badger M von Caemmerer S Ruuska S Nakano H Electron flow to oxygen in higher plants and algae: rates and control of direct photoreduction (Mehler reaction) and rubisco oxygenase Phil Trans R Soc Lond B 2000 355 1402 1433 1446 10.1098/rstb.2000.0704 11127997 PMC1692866

[B44] Hahn D Kaltenbach C Kück U The Calvin cycle enzyme sedoheptulose-1,7-bisphosphatase is encoded by a light-regulated gene in Chlamydomonas reinhardtii Plant Mol Biol 1998 36 6 929 934 10.1023/A:1005911022601 9520283

[B45] Xue X Gauthier D Turpin D Weger H Interactions between photosynthesis and respiration in the green alga Chlamydomonas reinhardtii: characterization of light-enhanced dark respiration Plant Physiol 1996 112 3 1005 1014 12226429 10.1104/pp.112.3.1005 PMC158027

[B46] Sueltemeyer D Klug K Fock H Effect of photon fluence rate on oxygen evolution and uptake by Chlamydomonas reinhardtii suspensions grown in ambient and CO2-enriched air Plant Physiol 1986 81 2 372 375 10.1104/pp.81.2.372 16664823 PMC1075342

[B47] Miura K Yamano T Yoshioka S Kohinata T Inoue Y Taniguchi F Asamizu E Nakamura Y Tabata S Yamato K Ohyama K Fukuzawa H Expression profiling-based identification of CO2-responsive genes regulated by CCM1 controlling a carbon-concentrating mechanism in Chlamydomonas reinhardtii Plant Physiol 2004 135 3 1595 1607 10.1104/pp.104.041400 15235119 PMC519074

[B48] Yamano T Miura K Fukuzawa H Expression analysis of genes associated with the induction of the carbon-concentrating mechanism in Chlamydomonas reinhardtii Plant Physiol 2008 147 1 340 354 10.1104/pp.107.114652 18322145 PMC2330288

[B49] Fang W Si Y Douglass S Casero D Merchant S Pellegrini M Ladunga I Liu P Spalding M Transcriptome-wide changes in Chlamydomonas reinhardtii gene expression regulated by carbon dioxide and the CO2-concentrating mechanism regulator CIA5/CCM1 Plant Cell 2012 24 5 1876 1893 10.1105/tpc.112.097949 22634760 PMC3442575

[B50] Winder T Anderson J Spalding M Transcriptional regulation of the large and small subunits of ribulose bisphosphate carboxylase/oxygenase during induction of the CO2-concentrating mechanism in Chlamydomonas reinhardtii Plant Physiol 1992 98 4 1409 1414 10.1104/pp.98.4.1409 16668808 PMC1080365

[B51] Heifetz P Förster B Osmond C Giles L Boynton J Effects of acetate on facultative autotrophy in Chlamydomonas reinhardtii assessed by photosynthetic measurements and stable isotope analyses Plant Physiol 2000 122 4 1439 1445 10.1104/pp.122.4.1439 10759539 PMC58978

[B52] Goldschmidt-Clermont M The two genes for the small subunit of RuBP Carboxylase/oxygenase are closely linked in Chlamydomonas reinhardtii Plant Mol Biol 1986 6 1 13 21 10.1007/BF00021302 24307150

[B53] Kroymann J Schneider W Zetsche K Opposite regulation of the copy number and the expression of plastid and mitochondrial genes by light and acetate in the green flagellate Chlorogonium Plant Physiol 1995 108 4 1641 1646 12228568 10.1104/pp.108.4.1641 PMC157545

[B54] Heifetz P Turpin D Gillham N Boynton J Osmond C dr and spr/sr mutations of Chlamydomonas reinhardtii affecting D1 protein function and synthesis define two independent steps leading to chronic photoinhibition and confer differential fitness Plant Cell Environ 1997 20 9 1145 1157 10.1046/j.1365-3040.1997.d01-143.x

[B55] Eppley R Gee R Saltman P Photometabolism of acetate by Chlamydomonas mundana Physiol Plant 1963 16 4 777 792 10.1111/j.1399-3054.1963.tb08355.x

[B56] Ball S Dirick L Martiat J Matagne R Physiology of starch storage in the monocellular alga Chlamydomonas reinhardtii Plant Sci 1990 66 1 1 9 10.1016/0168-9452(90)90162-H

[B57] Martinez-Rivas J Vega J Effect of culture conditions on the isocitrate dehydrogenase and isocitrate lyase activities in Chlamydomonas reinhardtii Physiol Plant 1993 88 4 599 603 10.1111/j.1399-3054.1993.tb01377.x 28741776

[B58] Chen F Johns M Substrate inhibition of Chlamydomonas reinhardtii by acetate in heterotrophic culture Process Biochem 1994 29 4 245 252 10.1016/0032-9592(94)80064-2

[B59] Fett J Coleman J Regulation of periplasmic carbonic anhydrase expression in Chlamydomonas reinhardtii by acetate and pH Plant Physiol 1994 106 1 103 108 12232308 10.1104/pp.106.1.103 PMC159504

[B60] Matsuo M Hachisu R Tabata S Fukuzawa H Obokata J Transcriptome analysis of respiration-responsive genes in Chlamydomonas reinhardtii: mitochondrial retrograde signaling coordinates the genes for cell proliferation with energy-producing metabolism Plant Cell Physiol 2011 52 2 333 343 10.1093/pcp/pcq192 21149298

[B61] Baurain D Dinant M Coosemans N Matagne R Regulation of the alternative oxidase Aox1 gene in Chlamydomonas reinhardtii. Role of the nitrogen source on the expression of a reporter gene under the control of the Aox1 promoter Plant Physiol 2003 131 3 1418 1430 10.1104/pp.013409 12644691 PMC166901

[B62] Finazzi G Johnson G Dall'Osto L Zito F Bonente G Bassi R Wollman F Nonphotochemical quenching of chlorophyll fluorescence in Chlamydomonas reinhardtii Biochemistry 2006 45 5 1490 1498 10.1021/bi0521588 16445291

[B63] Peers G Truong T Ostendorf E Busch A Elrad D Grossman A Hippler M Niyogi K An ancient light-harvesting protein is critical for the regulation of algal photosynthesis Nature 2009 462 7272 518 521 10.1038/nature08587 19940928

[B64] Bonente G Ballottari M Truong T Morosinotto T Ahn T Fleming G Niyogi K Bassi R Analysis of LhcSR3, a protein essential for feedback de-excitation in the green alga Chlamydomonas reinhardtii PLoS Biol 2010 9 1 e1000577 10.1371/journal.pbio.1000577 21267060 PMC3022525

[B65] Weger H Turpin D Mitochondrial respiration can support NO3- and NO2- reduction during photosynthesis. Interactions between photosynthesis, respiration and N assimilation in the N-limited green alga Selenastrum minutum Plant Physiol 1989 89 2 409 415 10.1104/pp.89.2.409 16666557 PMC1055855

[B66] Quesada A Hidalgo J Fernández E Three Nrt2 genes are differentially regulated in Chlamydomonas reinhardtii Mol Gen Genet 1998 258 4 373 377 10.1007/s004380050743 9648741

[B67] Gérin S Mathy G Blomme A Franck F Sluse F Plasticity of the mitoproteome to nitrogen sources (nitrate and ammonium) in Chlamydomonas reinhardtii: the logic of Aox1 gene localization BBA-Bioenergetics 2010 1797 6 994 1003 10.1016/j.bbabio.2010.02.034 20211595

[B68] Sakihama Y Nakamura S Yamasaki H Nitric oxide production mediated by nitrate reductase in the green alga Chlamydomonas reinhardtii: an alternative NO production pathway in photosynthetic organisms Plant Cell Physiol 2002 43 3 290 297 10.1093/pcp/pcf034 11917083

[B69] Camargo A Llamas Á Schnell R Higuera J González-Ballester D Lefebvre P Fernández E Galván A Nitrate signaling by the regulatory gene NIT2 in Chlamydomonas Plant Cell 2007 19 11 3491 3503 10.1105/tpc.106.045922 18024571 PMC2174885

[B70] Hyams J Davies D The induction an characterization of cell wall mutants of Chlamydomonas reinhardi Mutat Res 1972 14 4 381 389 10.1016/0027-5107(72)90135-2

[B71] Lowry O Rosebrough N Farr A Randall R Protein measurement with the Folin phenol reagent J Biol Chem 1951 193 1 165 175 14907713

[B72] Burnham K Anderson D Multimodel inference: understanding AIC and BIC in model selection Sociol Method Res 2004 33 2 261 304 10.1177/0049124104268644

[B73] Sall J Lehman A Stephens M Creighton L JMP Start Statistics: a Guide to Statistics and Data Analysis using JMP 2012

[B74] http://www.jmp.com/support/help/Regression_Reports.shtml **JMP. Fitting linear models. Standard least squares report and options. Regression reports.** []

