# Supplementary material for: Modeling the dependence of respiration and photosynthesis upon light, acetate, carbon dioxide, nitrate and ammonium in Chlamydomonas reinhardtii using design of experiments and multiple regression
Source: BMC Syst Biol. 2014 Aug 16;8:96. doi: 10.1186/s12918-014-0096-0 (PMC4236732; doi:10.1186/s12918-014-0096-0)
Supplement: Additional file 2 — Mathematical equations resulting from the 1st-round of modeling. These equations were obtained prior to the restriction of the models to the major explanatory factor(s) (as done in the 2nd-round of modeling). Due to its definition as an ordinal factor, CO2 concentration is present as an extension term which is equal to zero for 0.035% CO2 and different from zero for 1.5% CO2. [file s12918-014-0096-0-S2.pdf]

$$CR = 6.73 + 9.12\,[Acetate] + 0.0253\,Light - 0.433\,[{NH_4}^+] + 0.0227\,([Acetate] - 0.463)\,(Light - 108) - 0.556\,([Acetate] - 0.463)\,([{NH_4}^+] - 7.69)$$

$$MA_{CYT} = 6.55 + 7.64\,[Acetate] + 0.0255\,Light - 0.298\,[{NH_4}^+] - 0.0598\,[{NO_3}^-] + 0.0134\,([{NH_4}^+] - 7.13)\,([{NO_3}^-] - 9.51)$$
$$+ 0.0176\,([Acetate] - 0.500)\,(Light - 105)$$

$$MA_{ALT} = 10.6 + 3.69\,[Acetate] + 0.0207\,Light - 0.328\,[{NH_4}^+] + 0.0531\,[{NO_3}^-] - 13.3\,([Acetate] - 0.488)^2 - 0.367\,([Acetate] - 0.488)\,([{NH_4}^+] - 7.32)$$
$$+ 0.0180\,([{NH_4}^+] - 7.32)\,([{NO_3}^-] - 9.76) - 0.00125\,([{NH_4}^+] - 7.32)\,(Light - 107)$$

$$\Phi PSII_{800} = 0.361 + 0.0410\,[Acetate] + 0.00103\,Light - 0.000745\,[{NH_4}^+] - 0.246\,([Acetate] - 0.488)^2 - 0.0000428\,(Light - 107)\,([{NH_4}^+] - 7.32)$$
$$\boldsymbol{- 0.0348\ if\ [CO_2] = 1.5\%}$$

$$NPQ_{800} = 0.358 - 0.0862\,[Acetate] - 0.000153\,Light - 0.00328\,[{NH_4}^+] - 0.00138\,[{NO_3}^-] - 0.00110\,([{NO_3}^-] - 9.76)^2$$
$$- 0.000902\,([Acetate] - 0.488)\,(Light - 107)$$

$$P_{800} = 40.4 - 3.08\,[Acetate] + 0.187\,Light - 0.555\,[{NH_4}^+] + 0.882\,[{NO_3}^-] + 154\,([Acetate] - 0.488)^2 - 0.525\,([{NO_3}^-] - 9.76)^2$$
$$- 0.144\,([Acetate] - 0.488)\,(Light - 107) - 0.111\,([{NH_4}^+] - 7.32)\,(NO_3 - 9.76) + \boldsymbol{8.71\ if\ [CO_2] = 1.5\%}$$
