# Supplementary material for: Modeling the dependence of respiration and photosynthesis upon light, acetate, carbon dioxide, nitrate and ammonium in Chlamydomonas reinhardtii using design of experiments and multiple regression
Source: BMC Syst Biol. 2014 Aug 16;8:96. doi: 10.1186/s12918-014-0096-0 (PMC4236732; doi:10.1186/s12918-014-0096-0)
Supplement: Additional file 3 — β-weights, whole-model and individual effect ANOVA tests of the 2nd-round of modeling. Numbers ranging from 1 to 5 classify the different effects by increasing order of individual p-value. P-values which are surrounded by * are considered as statistically significant (p ≤ 0.05). [file s12918-014-0096-0-S3.pdf]

	**CR**		
	Whole-model ANOVA: *p*<0.0001*		
	Factor	***β***	***p-value***
1	[Acetate]	0.652	<0.0001*
2	$[NH_4^+]$	-0.466	<0.0001*
3	Light	0.374	<0.0001*
4	[Acetate]*$[NH_4^+]$	-0.297	0.0003*

	**MA_{CYT}**		
	Whole-model ANOVA: *p*<0.0001*		
	Factor	***β***	***p-value***
1	[Acetate]	0.602	<0.0001*
2	Light	0.419	0.0001*
3	$[NH_4^+]$	-0.337	0.0015*

	**MA_{ALT}**		
	Whole-model ANOVA: *p*<0.0001*		
	Factor	***β***	***p-value***
1	$[NH_4^+]$	-0.450	<0.0001*
2	Light	0.395	0.0004*
3	[Acetate]	0.344	0.0016*
4	[Acetate]*[Acetate]	-0.296	0.0058*
5	[Acetate]*$[NH_4^+]$	-0.263	0.0127*

	**$\Phi PSII_{800}$**		
	Whole-model ANOVA: *p*<0.0001*		
	Factor	***β***	***p-value***
1	Light	0.810	<0.0001*

	**NPQ_{800}**		
	Whole-model ANOVA: *p*=0.0002*		
	Factor	***β***	***p-value***
1	$[NO_3^-]$*$[NO_3^-]$	-0.414	0.0016*
2	[Acetate]*Light	-0.366	0.0043*
3	[Acetate]	-0.360	0.0049*
4	$[NO_3^-]$	-0.120	0.3260
5	Light	-0.120	0.3280

	**P_{800}**		
	Whole-model ANOVA: *p*<0.0001*		
	Factor	***β***	***p-value***
1	Light	0.572	<0.0001*
2	$[NO_3^-]$*$[NO_3^-]$	-0.875	0.0002*
3	[Acetate]*[Acetate]	0.657	0.0034*
4	$[NO_3^-]$	0.272	0.0145*
5	[Acetate]	-0.049	0.6448
